# Polymorphism and flexibility of six-porphyrin nanorings in the solid state†

**DOI:** 10.1039/d4sc05255b

**Published:** 2024-09-18

**Authors:** Wojciech Stawski, Harry L. Anderson

**Affiliations:** a Department of Chemistry, University of Oxford, Chemistry Research Laboratory Oxford OX1 3TA UK harry.anderson@chem.ox.ac.uk w.stawski97@gmail.com

## Abstract

Butadiyne-linked porphyrin nanorings are fascinating nanometer-sized platforms for exploring electronic delocalization and aromaticity, and they mimic ultra-fast photosynthetic energy-transfer phenomena in plants and purple bacteria. However, little is known about how they interact in the solid state. Here, we compare the crystal structures of several pseudopolymorphs of a six-porphyrin nanoring template complex, and report the structure of the free-base nanoring co-crystallized with C_60_. The structures differ not only in the molecular packing; they also feature different molecular conformations. The template is slightly too small for the cavity of the nanoring, and this size mismatch can be accommodated by two types of distortion: either the zinc atoms are pulled away from the planes of the porphyrins, or the nanorings contract by adopting a ruffled conformation, with butadiyne links alternatingly above and below the plane of the six zinc centers. The template-bound ring forms sheets and tubular stacks with interdigitated aryl groups. Upon demetallation, the nanoring becomes more flexible, adopting a highly elliptical conformation on co-crystallization with C_60_. The structure of this free-base nanoring features infinite solvent filled channels with a channel diameter of 13.5 Å. The high porosity of these materials points towards possible applications as porous light-harvesting frameworks.

## Abstract 2

The template complex of a zinc porphyrin nanoring forms several pseudopolymorphs featuring different conformations of the π-system. The metal-free nanoring is even more flexible, and adopts an elliptical geometry when co-crystallized with C_60_.

## Graphical abstract

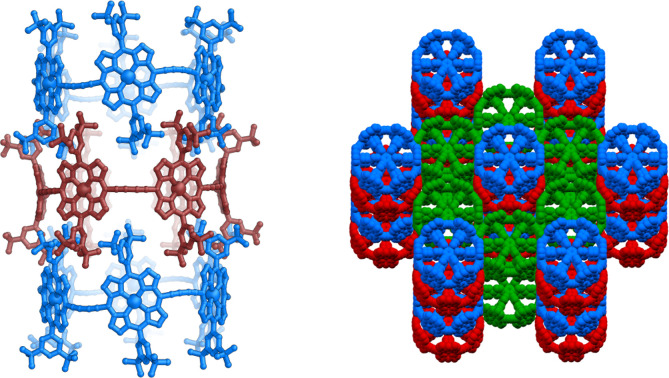

## Introduction

π-Conjugated cyclic porphyrin oligomers (porphyrin nanorings) with butadiyne linkers^1–4^ have been investigated extensively to probe cooperativity in multivalent molecular recognition,^2^ test the size limit of aromaticity^3^ and provide models for ultrafast energy migration in photosynthesis.^4^ Accurate crystallographic molecular geometries are valuable for analyzing host–guest interactions and for understanding aromaticity,^5^ yet only two crystal structures of these porphyrin nanorings have been previously reported.^6,7^

**Fig. 1:**
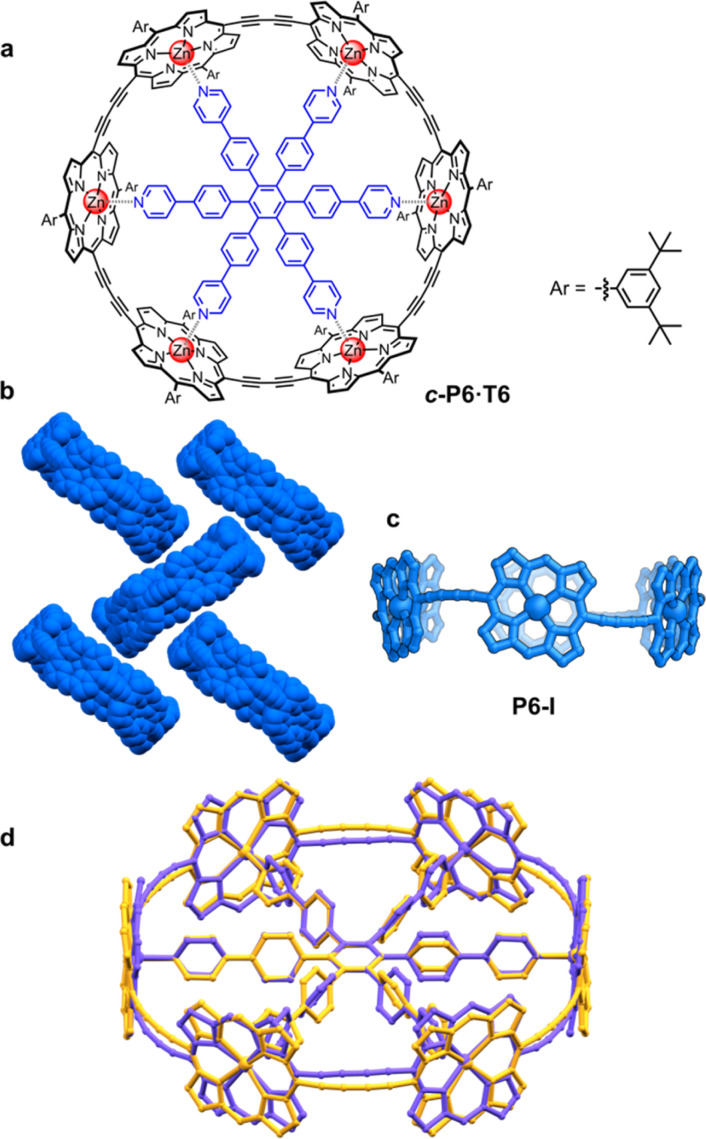
(a) Drawing of a six-porphyrin nanoring with the template marked in blue, Ar = 3,5-di-*tert*-butylphenyl. (b) Fragment of the crystal structure of the previously reported, orthorhombic (*Pccc*) polymorph P6-I (CCDC: 860774).^6^ (c) View of the molecular conformation of the nanoring in P6-I. Hydrogen atoms, template and aryl groups in (b) and (c) are omitted for clarity. (d) Comparison of the molecular geometry of *c-*P6·T6 from the published crystal structure of polymorph P6-I (purple)^6^ and the gas-phase geometry from DFT calculations (BLYP35/6-31G*, yellow).^16^

Based on the symmetry of the six-porphyrin nanoring *c-*P6·T6 (Fig. 1a), it might be expected to form hexagonal arrays, resulting in tubular stacks and infinite 2D sheets. However, the crystal structure reported previously for this nanoring complex, which we call here the P6-I polymorph, exhibits non-porous herringbone-type packing (Fig. 1b).^6^ Porous organic frameworks are widely investigated for applications such as gas storage and sensing,^8^ and many macrocycles have been reported to form porous crystals,^9^ which suggests that porphyrin nanorings might exhibit this type of behavior. However, macrocycles such as cycloparaphenylenes (CPPs),^10^ cucurbiturils and cyclodextrins^11^ often adopt compact non-porous herringbone packing arrangements. In the whole family of the CPPs, only one member, [6]CPP, has been found to crystallize in a porous tubular arrangement.^12^ In this case, the tubular stacks can be reinforced by intermolecular C–H⋯F interactions, after partial substitution with fluorine.^13^ The confined spaces created by this type of packing have been used to form host–guest composites.^14^ Here we report the structures of new pseudopolymorphs of *c-*P6·T6 that have large solvent-accessible voids. (These structures of *c-*P6·T6 are described as pseudopolymorphs, rather than polymorphs, because they differ in solvent composition.^15^)

A curious feature of the P6-I structure is that the butadiyne linkers are substantially curved above and below the mean plane of the six zinc atoms (Fig. 1c), in a pattern reminiscent of a cyclohexane chair. This distortion is attributed to the fact that the template is too small for the cavity of the nanoring (by *ca.* 2.5%), but, surprisingly, the gas-phase geometry of *c*-P6·T6, calculated using density functional theory (DFT),^16^ has a different structure, with the butadiyne-linkers in the plane of the zinc atoms. The difference between these two structures is seen from the overlay in Fig. 1d. The nanoring seems to have two ways to contract around the template: (a) tilting the porphyrins to ruffle the perimeter, displacing the butadiynes out of plane, as in P6-I, or (b) bending the porphyrin skeleton to pull the Zn ion away from the porphyrin mean plane, as in the gas-phase structure. The new pseudopolymorphs of *c-*P6·T6, reported here, provide insight into the tension between these two distortions.

During crystallization of samples of *c-*P6·T6, over a decade ago, besides the crystals that gave the published structure P6-I, it was reported that a second pseudopolymorph was persistently observed with hexagonal symmetry.^6^ The structure of this other pseudopolymorph could not be determined because the lack of long-range order in one direction resulted in essentially no diffraction at some orientations of the crystal. However, the data collected at that time were sufficient to determine unit cell dimensions (*a*, *b* = 49.85 Å, *c* = 29.46 Å *α* = *β* = 90°, *γ* = 120°). These parameters, and the behavior of the crystals during diffraction experiments, suggested formation of flat layers of nanorings with hexagonal symmetry, separated by disordered solvent.

Scanning tunneling microscopy (STM) provides some insights into the packing behavior of porphyrin nanorings. Template-free nanorings (*c-*P*N*s) with *N* > 8 form highly ordered arrays on a graphite surface, with the porphyrins parallel to the surface. The larger nanorings (*N* = 24–40) also form stacked aggregates on a gold surface, if deposited in the absence of pyridine.^17^ However, STM images only provide information in two dimensions and lack atomic resolution. Moreover, smaller rings such as *c-*P6 do not lie flat on the surface because they are cylindrical, making them difficult to study by STM. We decided to perform a thorough crystallographic study of these nanorings to gain insights into how they pack in the solid state, choosing the six-porphyrin ring *c-*P6 as a suitable candidate due to its high symmetry and synthetic accessibility.^6^

## Results and discussion

### Analysis of the previously reported structure P6-I

**Fig. 2:**
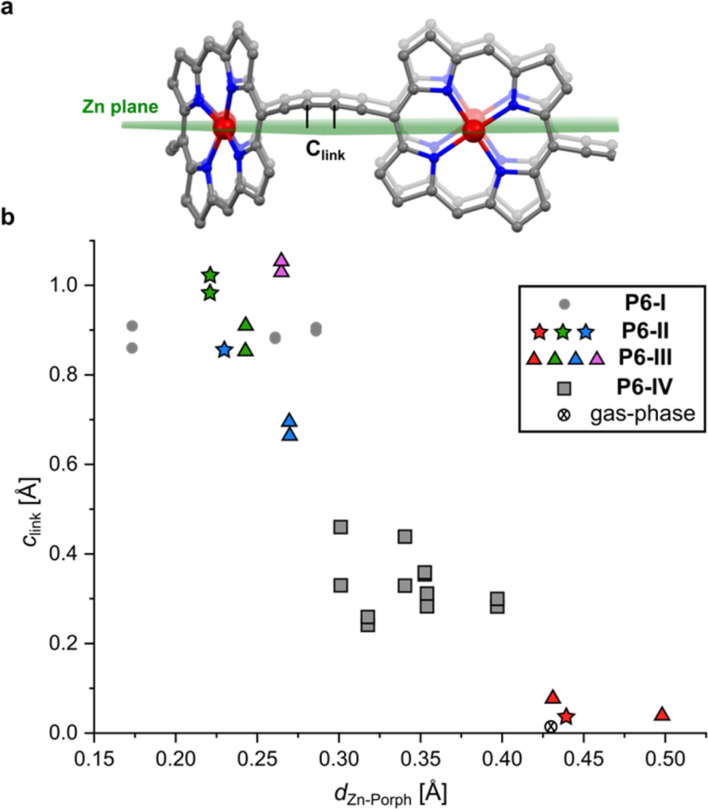
(a) Illustration showing the mean plane of the six Zn atoms and the distance of the sp-hybridized carbon atoms of the butadiyne linkers to this plane (*c*_link_); (b) relationship between the Zn-to-porphyrin-plane distance (*d*_Zn–Porph_) and the out-of-Zn plane distance of the carbon atoms in the butadiyne linkers (*c*_link_) in the four pseudopolymorphs. The colors of the dots correspond to the colors of the nanorings in Fig. 3 (P6-II and P6-III), with gray squares corresponding to the P6-IV polymorph. The mean porphyrin plane was calculated from the coordinates of all 24 non-H atoms from the porphyrin core. Data for the DFT-optimized model (BLYP35 6-31G*)^16^ were averaged from six porphyrin rings and 12 carbon atoms from the linkers for clarity.

The difference between structure P6-I and the gas-phase DFT structure of *c-*P6·T6 can be viewed by plotting the distance of the two central carbon atoms of the butadiyne units from the mean plane of the six zinc centers (*c*_link_, defined in Fig. 2a) against the Zn to porphyrin mean-plane distance (*d*_Zn–Porph_). The gray circles in Fig. 2b show that P6-I has a long *c*_link_ = 0.89 Å and a short out-of-plane distance, *d*_Zn–Porph_ = 0.24 ± 0.06 Å, whereas the cross denoting the gas-phase DFT structure has *c*_link_ ≈ 0 and *d*_Zn–Porph_ = 0.43 Å. Here we present the structures of three more pseudopolymorphs of *c-*P6·T6 (P6-II, P6-III and P6-IV; Table 1) revealing that the gas-phase structure and P6-I are extreme cases. The other pseudopolymorphs of *c*-P6·T6 exhibit a continuous spectrum of both types of distortion (Fig. 2b).

**Table 1:** Space group and unit cell parameters of the crystal, measured at temperature *T* = 100 K (or 150 K for P6-I only)

Crystal	S.G.^a^	*a*, *b*, *c* [Å]	*α*, *β*, *γ*	*V* [Å^3^]
P6-I^6^	*Pccc*	59.310(5)	90°	46 229(9)
		25.141(3)	90°	
		31.050(4)	90°	
P6-II	*P*3̄*m*1	49.3794(3)	90°	60 585.6(11)
		49.3794(3)	90°	
		28.6911(5)	120°	
P6-III	*P*3̄*m*1	49.7450(2)	90°	118 348.4(12)
		49.7450(2)	90°	
		55.2247(4)	120°	
P6-IV	*P*1̄	20.6135(2)	81.878(1)°	29 535.9(5)
		38.7108(3)	80.210(1)°	
		39.1085(4)	74.897(1)°	
P6-C_60_	*I*2/*m*	20.1388(3)	90°	32 380.1(8)
		53.4956(7)	106.792(1)°	
		31.8306(4)	90°	

a Space group symbol (in the Hermann–Mauguin notation).

### Crystallization experiments

The molecular weight of *c*-P6·T6 (5.8 kDa) is similar to that of a small protein such as insulin, and its crystallization and structure determination pose several challenges: (a) loss of solvent from large voids results in the loss of crystallinity, (b) the large unit cell reduces the achievable diffraction intensity and resolution, (c) disorder in the crystals reduces the quality of the X-ray diffraction data. We mitigated these problems to some extent by using a high-flux X-ray source (rotating anode) and choosing a non-volatile solvent for crystallization (*o*-dichlorobenzene). Additionally, we used 3,5-di-*tert*-butylphenyl solubilizing groups to confer solubility and crystallinity. After removing the crystals from the mother liquor, they must be manipulated extremely quickly (within seconds), otherwise they start dissolving in all the cryoprotecting oils that we have tested (Fig. S4†). Large solvent-accessible voids containing diffuse electron density from highly disordered solvent molecules made it necessary to use masking procedures (Olex2's implementation of BYPASS).^18^ The resolution and data quality do not allow detailed analysis of bond lengths and angles (due to the issues mentioned above), but the data are sufficient to establish the molecular conformation, connectivity and packing.

We grew crystals of three pseudopolymorphs of *c*-P6·T6: trigonal P6-II and P6-III, and triclinic P6-IV, followed by a monoclinic C_60_ cocrystal of the free-base ring P6-C_60_. All crystals were grown by diffusion of methanol into solutions in *o*-dichlorobenzene. P6-III and P6-IV crystallize together, whereas crystallization of P6-II was induced by addition of a small amount of 1,4-diazabicyclo[2.2.2]octane (DABCO) to the solution. DABCO was not found in the crystal structure, (as confirmed by ^1^H NMR analysis of dissolved crystals; see Fig. S36 and S37†), but its presence facilitates crystallization of this form. The unit cell parameters for all the crystals are summarized in Table 1.

### Structure P6-II

**Fig. 3:**
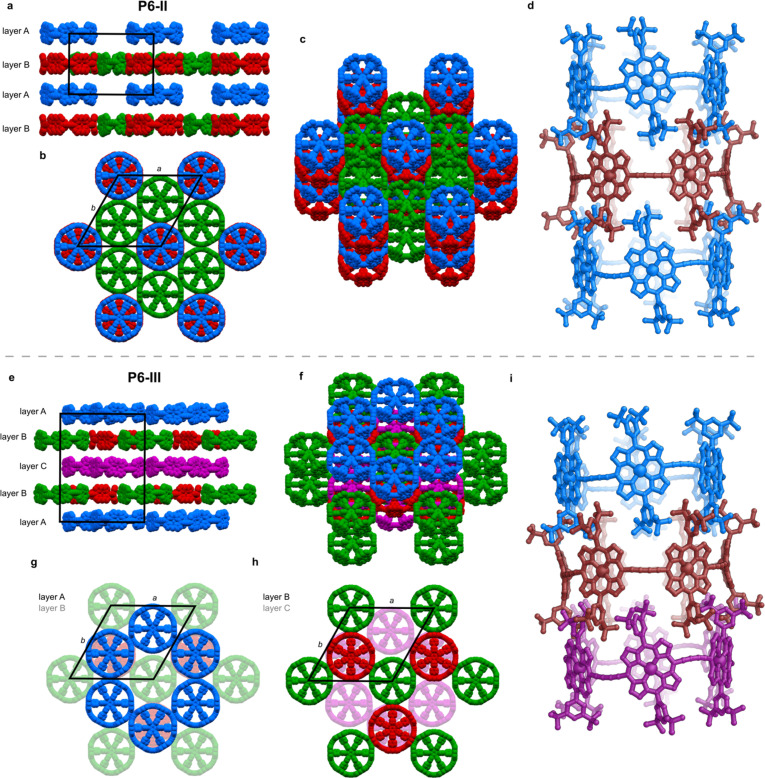
Crystal structures of P6-II and P6-III. Different colors indicate crystallographically independent molecules and unit cell edges are indicated as black lines. P6-II: (a) view along *a* axis; (b) view along *c* axis; (c) skewed view on the structure; (d) view on part of the infinite columnar stack with template removed for clarity. P6-III: (e) view along *a* axis; (f) side view; (g and h) view on the layers from the *c* direction, with the bottom layers less opaque; (i) view on a tubular stack of the rings (template removed for clarity).

The unit cell of the trigonal pseudopolymorph P6-II (Table 1, second entry) is similar to that of the unsolved hexagonal crystals reported in 2011 (ref. 6) (*a*, *b* = 49.85 Å, *c* = 29.46 Å). We probably observe the same type of packing, with small differences related to the solvents used for crystallization (chloroform and *n*-hexane were used previously), which also results in a different crystal morphology (brown disks for P6-II, instead of black hexagonal plates). In structure P6-II, there are three crystallographically independent molecules of *c*-P6·T6 forming two distinct layers, assigned as A and B (Fig. 3a).

Layer A consists of nanoring molecules (blue in Fig. 3) surrounded by nanoring-sized vacancies. In layer B, red and green molecules form pseudo six-fold symmetric sheets in the *ab* plane, each ring being surrounded by six others: red ones by six green molecules, and each green one by three green and three red rings (Fig. 3b and c). The green rings have short butadiyne–butadiyne contacts, whereas between the red and green molecules there are short porphyrin–butadiyne contacts (Fig. S8–S11†). The blue ones pack above red rings, and together they form infinite tubular stacks along the *c* axis, with aryl groups of each ring interdigitating between the aryls of another ring (Fig. 3d). These infinite tubular stacks of red and blue nanorings are partly interrupted by template units. There are also solvent-accessible channels running through the green nanorings and between the nanorings (Fig. S26†).

The three crystallographically independent nanorings in P6-II differ in their molecular geometries. The blue and green ones have substantial curving of the butadiyne linkers and porphyrinic walls are tilted to the side, as in P6-I.^6^ The porphyrin units in the blue and green nanorings are planar (root-mean-square deviation, RMSD, of the 24-atom mean porphyrin plane of 0.046 and 0.062 Å, respectively), with zinc(ii) ions only slightly displaced from the plane (*d*_Zn–Porph_ = 0.23 and 0.22 Å) and the distance of carbon atoms in the butadiyne linkers from the mean six-Zn plane is *c*_link_ = 0.86–1.02 Å, as shown in Fig. 2b.^19^

The linkers in the red rings lie approximately in the Zn plane (*c*_link_ = 0.04 Å) and the porphyrins bend outwards (RMSD 0.204 Å), exposing the zinc(ii) centers above the porphyrin mean plane (*d*_Zn–Porph_ = 0.44 Å), similar to the gas-phase calculated geometry.

### Structure P6-III

In the second trigonal pseudopolymorph P6-III, the length of the unit cell edge *a* is close to that of P6-II, but the *c* parameter is roughly twice as long. In contrast to P6-II, as reflected by in the unit cell dimensions, there are three distinct layers (A, B and C; Fig. 3e–i) occupied in total by four crystallographically independent nanorings. The main difference with respect to P6-II is that in each layer of P6-III, six rings (six blue or purple in layers A/C and three red and three green in layer B) surround nanoring-sized voids. Thus, the nanorings do not form infinite tubular stacks in the *c*-direction. Instead, the blue, red and purple rings for trimeric stacks (Fig. 3i), separated by the vacancies surrounded by red and green rings. Three distinct molecules adopt wavy conformations (*c*_link_ = 0.67–1.06 Å), whereas in the fourth (red) the butadiyne linkers lie approximately in the Zn atom plane, similar to the gas-phase geometry (*c*_link_ = 0.04–0.08 Å), as illustrated in Fig. 2b. In parallel with P6-II, the only intermolecular butadiyne–porphyrin contacts are between the red (linkers in the Zn plane) and green rings (wavy linkers) and the contacts between blue rings and also between the violet rings are butadiyne-to-butadiyne (Fig. S14–S16†).

### Structure P6-IV

**Fig. 4:**
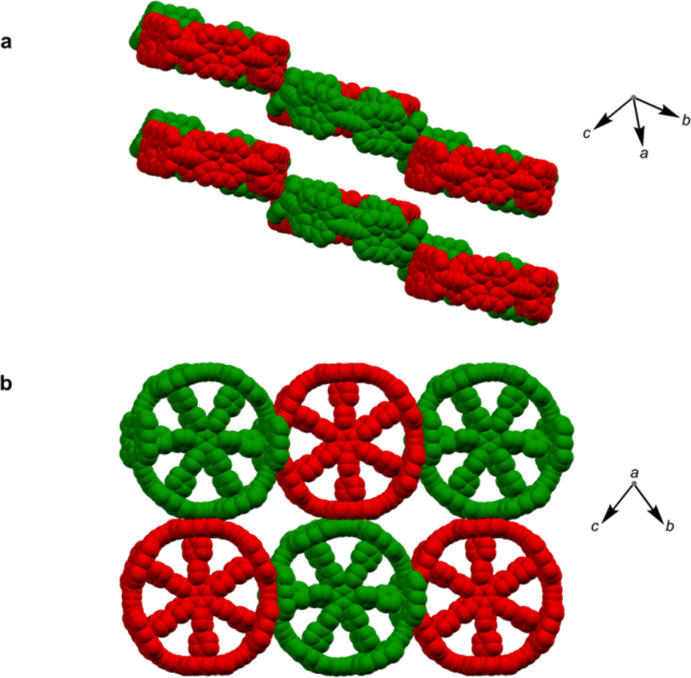
Two orthogonal views on the packing in the triclinic polymorph P6-IV. (a) View on the layers in the crystal structure; (b) view on one of the layers. Hydrogen atoms and aryl groups were omitted for clarity. Two colors indicate crystallographically independent molecules.

The third pseudopolymorph P6-IV with triclinic symmetry (Table 1, fourth entry) crystallizes as brown plates containing two crystallographically independent molecules forming layers (Fig. 4). The nanorings are slipped with respect to each other (Fig. S19†). The butadiyne linkers adopt wavy geometries, although the twisting is observed to a smaller extent than in P6-II and P6-III and *c*_link_ does not exceed 0.44 Å. The RMSD of porphyrin planes is 0.070–0.165 Å and the Zn ions are significantly displaced from the porphyrin plane (*d*_Zn–Porph_ = 0.30–0.40 Å). The rings are arranged in a porphyrin–butadiyne stacking manner (Fig. S20–S21†).

All the pseudopolymorphs P6-I–P6-IV are highly solvated. In P6-II, P6-III and P6-IV, the solvent-accessible voids (probe radius 1.2 Å, calculated with Mercury 4.0 software^20^ after removing the refined solvent molecules) constitute between 28% and 33% of the unit cell volume (Fig. S26–S28†). On the other hand, the herringbone-type packing in P6-I reduces the void volume by more than a factor of two (13%, see Fig. S25†).

### Structure P6-C_60_

All the structures discussed above are for the zinc nanoring *c*-P6 coordinated to the T6 template. The presence of the bound template, and coordination to metal cations, increases the rigidity of this nanoring complex. Now we turn to the free-base nanoring with no template, which is expected to be more flexible. Porphyrins were extensively used by Balch, Olmstead, and coworkers as co-crystallization partners to obtain crystal structures of functionalized fullerenes.^21^ Here we use the reverse strategy to facilitate crystallization of porphyrin nanorings, taking inspiration from the many reported fullerene–porphyrin nanostructures.^14*a*,22^ We hypothesized that π-stacking of fullerene molecules to the porphyrinic walls could govern their assembly into 2D sheets.

**Fig. 5:**
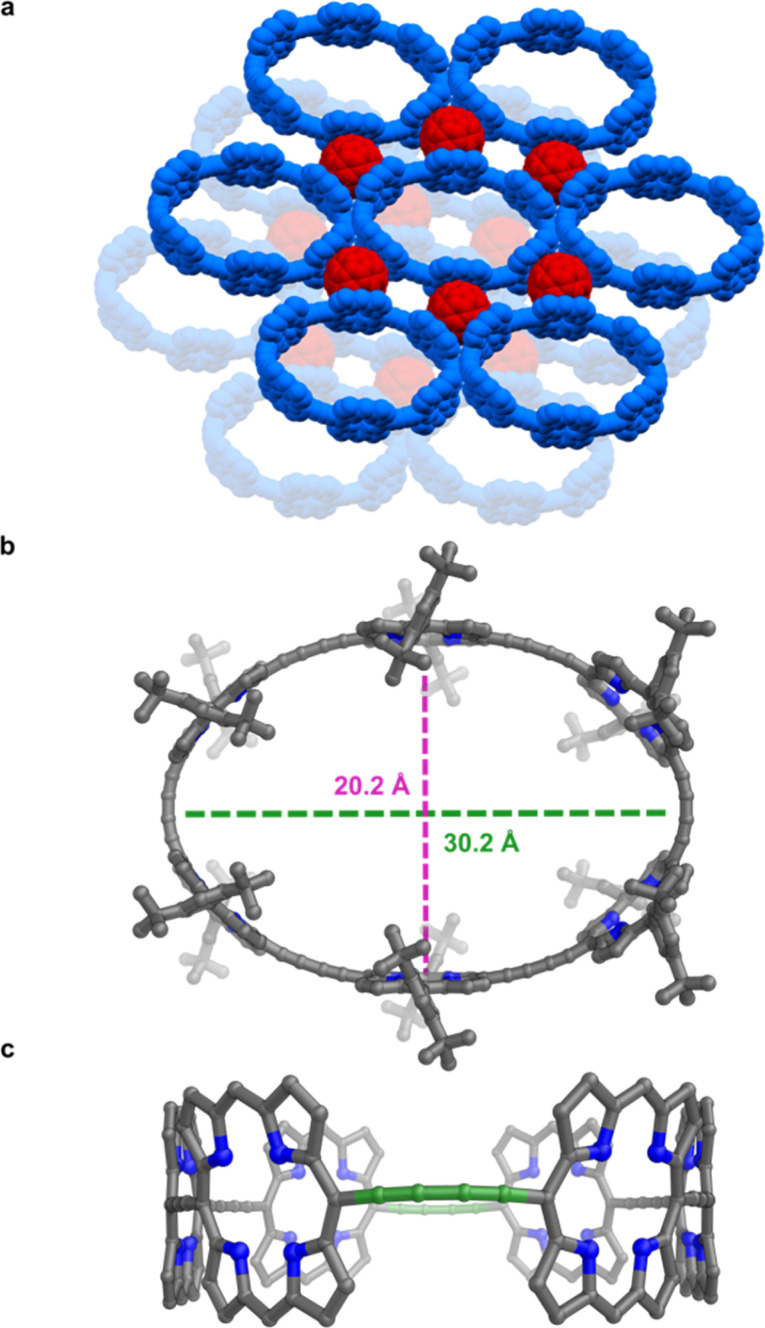
(a) Packing in P6-C_60_, with the bottom layer in the crystal structure shown with lower opacity; (b) view on the nanoring molecule with indicated ellipse axes' lengths; (c) side-view showing two opposite, bent butadiyne linkers (shown in green).

Vapor diffusion of methanol into a solution of free-base nanoring in *o*-dichlorobenzene containing 2.0 equiv. of C_60_ provided P6-C_60_ as dark green blocks. Each nanoring is surrounded by six fullerenes (Fig. 5a) which stack to the porphyrin walls, forming continuous layers. The rings in the different layers are shifted with respect to each other (unlike in P6-II and P6-III). The most striking feature is the geometry of the nanoring itself: it is highly elliptical (major axis: 30.2 Å, minor axis: 20.2 Å; Fig. 5b), with two butadiyne bridges slightly bent (largest *c*_link_ 0.18 Å)^23^ and the others in plane (largest *c*_link_ 0.01 Å; Fig. 5c), reflecting the surprising flexibility of the metal-free ring compressed in between fullerenes. In contrast, GFN2-xtb^24^ calculations for the free-base ring in the gas phase indicate that the lowest energy conformation is circular (Fig. S42†). The bending of the ring in the solid state to an elliptical conformation is evidently caused by interactions with fullerenes and other crystal-packing effects. The model shows short porphyrin centroid–fullerene 6 : 6 ring juncture contacts, as commonly observed for cocrystals with porphyrins,^25^ with a shortest contact of 2.772(7) Å between the centroid of the 6 : 6 ring juncture and the centroid of the 24-atom porphyrin plane (Fig. S24†). In contrast to the crystals of P6-II, P6-III and P6-IV, those of P6-C_60_ are difficult to re-dissolve, reflecting the strength of the crystal packing and interactions between fullerene molecules and the nanorings. There have been few reported cyclic fullerene receptors with three porphyrin walls surrounding fullerene molecules,^26^ and none of them were characterized by single-crystal X-ray crystallography. The Cambridge Structural Database contains only a few examples of a fullerene molecule stacking to three porphyrins.^27^

**Fig. 6:**
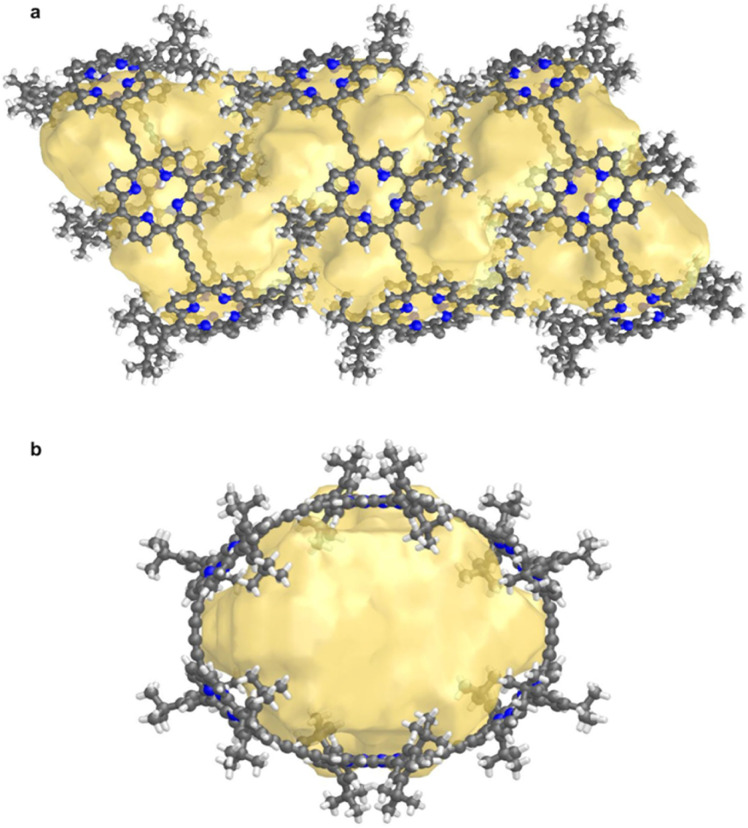
Visualization of the solvent-accessible channel in P6-C_60_. (a) view perpendicular to the channel, and (b) view along *a* axis, along the direction of the channel. Solvent accessible void calculations were performed using CageCavityCalc (C3) software^28^ using a spherical probe radius of 1.2 Å, based on the structure in which all solvent molecules had been removed.

Structure P6-C_60_ is highly porous and solvent-accessible voids constitute 40% of the unit cell volume (calculated with Mercury 4.0 software,^20^ probe radius 1.2 Å, after removing refined solvent molecules). This structure features infinite channels running though the solvent-filled cavities of the nanorings, along the direction of the *a* axis, as shown in Fig. 6.^28^ We analyzed these channels using the software tool Zeo++^29^ which gave a largest free sphere diameter of 13.5 Å; this is the largest free sphere that can move unimpeded between the periodic repeats of the channel. The largest included sphere diameter is 16.8 Å (both calculated using a probe radius of 1.2 Å).

## Conclusions

Single crystal X-ray diffraction analysis has revealed that butadiyne-linked six-porphyrin rings form hexagonal arrays, tubular stacks with interdigitated aryl groups and fullerene cocrystals. Complex molecular compounds often form multiple pseudopolymorphs.^30^ Thus it is not surprising that the *c-*P6·T6 nanoring complex forms pseudopolymorphs with different packing arrangements, but it is amazing that four of these pseudopolymorphs could be structurally characterized, without using a synchrotron X-ray source, considering that this complex has a molecular weight of 5.8 kDa and that the crystals contain up to four crystallographically independent *c-*P6·T6 units, together with large quantities of disordered solvent. This result highlights recent advances in X-ray crystallography. In contrast, the structure of the first pseudopolymorph P6-I, reported in 2011,^6^ was only determined after many data sets had been acquired at synchrotrons (both the Daresbury Synchrotron Radiation Source and the Diamond Light Source).

The spectrum of conformations adopted by the *c-*P6 nanoring in these pseudopolymorphs (Fig. 2b) reflects the different ways of balancing the tension between two types of molecular distortion. It is particularly interesting that the ruffled conformation of the nanoring is not reproduced by gas-phase DFT calculations.

These π-conjugated nanorings are shape-persistent, yet surprisingly flexible. The most dramatic illustration of this flexibility is the elliptical geometry of the free-base nanoring co-crystallized with C_60_. Porphyrin nanorings combine the flexibility of the individual porphyrin subunits^31^ and butadiyne linkers.^32^

Several of the structures presented here feature large solvent-accessible voids, particularly the P6-C_60_ structure, which contains infinite solvent-filled channels (largest free sphere diameter 13.5 Å). None of these structures can be strictly described as ‘porous crystals’ because the channels are filled with solvent, and so far our attempts at removing the solvent have resulted in loss of crystallinity. Capillary pressure arising from loss of solvent from a porous material can cause the pores to collapse, and this problem can often be avoided using supercritical solvents.^33^ In future work, we plan to exchange the solvent in P6-C_60_ with supercritical CO_2_ and to test whether this allows desolvation without collapse. The strong interactions between the porphyrins and C_60_, which make crystals of P6-C_60_ slow to redissolve, may stabilize the solvent-free lattice. It would also be interesting to explore whether excited state energy migration in crystals of nanorings mimics the behavior of light harvesting chlorophyll arrays,^34^ and whether porous crystals of nanorings can be applied as photocatalysts.^35^

## Data availability

The data that support the findings of this study are available in the ESI† of this article. Crystallographic data have been deposited at the Cambridge Crystallographic Data Centre. Deposition numbers: 2374893 (P6-II) 2374895 (P6-III), 2374896 (P6-IV) and 2374894 (P6-C_60_).

## Author contributions

W. S. had the idea for the project, synthesized the nanorings, performed crystallization, X-ray diffraction measurements, solved and refined structures and carried out theoretical calculations. The draft manuscript was written by W. S. and edited by H. L. A.

## Conflicts of interest

There are no conflicts to declare.

## Supplementary Material

SC-OLF-D4SC05255B-s001

SC-OLF-D4SC05255B-s002
