# Supplementary material for: Polymorphism and flexibility of six-porphyrin nanorings in the solid state
Source: Chem Sci. 2024 Sep 18;15(41):16938–46. doi: 10.1039/d4sc05255b (PMC11421218; doi:10.1039/d4sc05255b)
Supplement: SC-015-D4SC05255B-s001 [file SC-015-D4SC05255B-s001.pdf]

Supplementary Information (SI) for Chemical Science.
This journal is © The Royal Society of Chemistry 2024

Electronic Supporting Information

Polymorphism and flexibility of six-porphyrin nanorings in the solid state

Wojciech Stawski*[a] and Harry L. Anderson*[a]

a Department of Chemistry, University of Oxford
Chemistry Research Laboratory, Oxford OX1 3TA (UK)
E-mail: harry.anderson@chem.ox.ac.uk; w.stawski97@gmail.com

Table of Contents

1. General methods S2
2. Synthesis and crystallization S2
3. Measurement and refinement details S4
4. Crystal structure analysis S12
5. NMR and MS spectra S32
6. Theoretical calculations S34
7. References S40

1. General Methods

Solvents and reagents were purchased from Sigma Aldrich and used without further purification. ^{1}H NMR spectra were recorded on Bruker AVIII HD 400 MHz NMR spectrometer and were referenced against the residual solvent peak ($CDCl_3$, δ_H = 7.26 ppm). MALDI-TOF mass spectrum was recorded using a Bruker Autoflex instrument with DCTB as a matrix, depositing solution in dichloromethane on a plate and allowing it to get dry. Reflectron mode was used.

X-ray data collections for **P6-II**, **P6-III** and **P6-IV** was performed at the Department of Chemistry, University of Oxford using Rigaku Synergy DW diffractometer equipped with rotating anode source and HyPix-Arc 150° detector at 100 K and subsequently reduced using CrysAlisPro. After transferring suspension of crystals in mother liquor to Paratone oil (or NVH oil) smeared over a glass slide, suitable crystal was very quickly (few seconds) placed on a 50 µm MiTeGen loop and mounted on a goniometer head. Data collection for **P6-C$_{60}$** was performed at the Department of Chemistry, University of Oxford using Rigaku Super Nova A diffractometer at 100 K and then reduced using CrysAlisPro. Crystals were manipulated similarly as described above and mounted using a 200 µm MiTeGen loop.

2. Synthesis and crystallization

The template complex of the six-porphyrin nanoring ***c*-P6·T6** was synthesized from the corresponding porphyrin monomer as reported previously in 10% yield.[1]

Synthesis of the free-base nanoring *c*-P6-free

***c*-P6·T6** (10 mg, 1.7 µmol, 1 equiv.) was dissolved in dichloromethane (10 mL) and concentrated hydrochloric acid (0.1 mL, 1.16 mmol, 680 equiv.) was added. The solution was stirred vigorously for 15 min, followed by addition of saturated solution of $NaHCO_3$ (20 mL). A color change from brown to green was observed. Extraction was performed, adding additional portion of dichloromethane (10 mL). The organic layer was separated and dried over anhydrous Na_2SO_4, followed by removal of the solvent using a rotary evaporator. Next, the template was removed by performing size-exclusion chromatography (Bio-Beads S-X1) in THF, collecting the intense green band. The solvent was removed and the pure product was precipitated on a rotary evaporator with methanol to remove the THF stabilizer (BHT, butylated hydroxytoluene). Yield: 6.0 mg (80%).

^{1}H NMR (400 MHz, $CDCl_3$) δ 9.60 (d, *J* = 4.7 Hz, 24H, β_H), 8.79 (d, *J* = 4.7 Hz, 24H, β_H), 7.95 (d, *J* = 1.8 Hz, 24H, *m*-Ar), 7.79 (t, *J* = 1.8 Hz, 12H, *o*-Ar), 1.50 (s, 228H, *t*Bu), -1.34 (br. s, 12H, NH).

MS (MALDI) *m/z*: $[M]^+$ calcd for $C_{312}H_{312}N_{24}$ 4397.5; found: 4393.8.

Crystallization of P6-II

c-P6·T6 (1.5 mg, 0.26 µmol) was dissolved in *o*-dichlorobenzene (0.5 mL) containing DABCO (0.06 mg, 0.52 µmol, 2.0 equiv.) and the solution was layered with methanol (1.5 mL) in a vial. After four days at 20 °C the layers were fully mixed and brown, disk-shaped crystals of **P6-II** appeared on the walls, together with amorphous precipitate at the bottom of the vial.

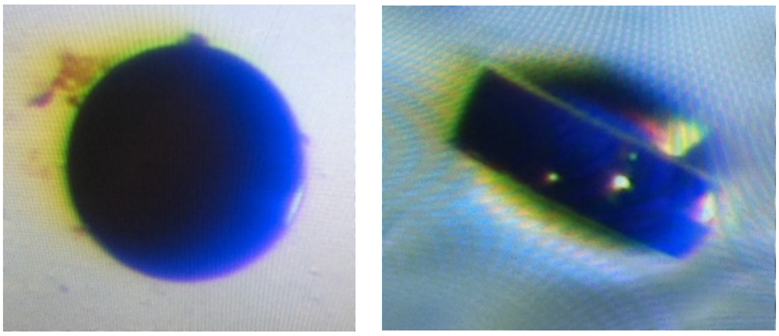

Figure S1. Crystal of **P6-II** photographed in two orientations. The crystal diameter is 50 µm.

Crystallization of P6-III and P6-IV

Nanoring **c-P6·T6** (1.5 mg, 0.26 µmol) was dissolved in *o*-dichlorobenzene (0.15 mL) and placed in a small vial inside a larger vial filled with methanol, which was closed. After one week at 20 °C, the solution became almost colorless, and crystals were formed: tear-shaped **P6-III** and plate-like **P6-IV**, both brown with green shine.

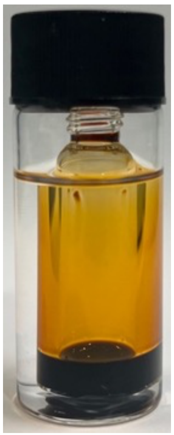

Figure S2. Crystallization set up for **P6-III** and **P6-IV**.

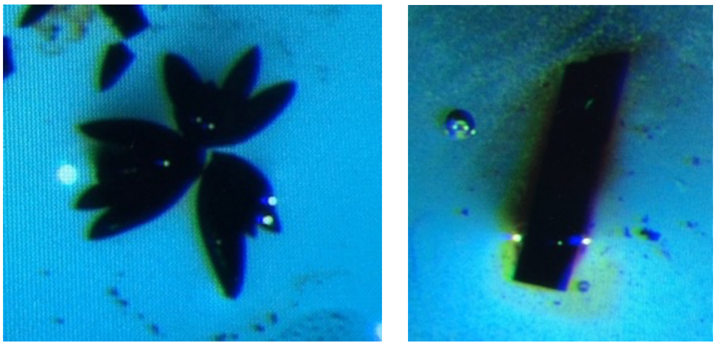

Figure S3. Crystals of **P6-III** (left) and **P6-IV** (right). Thickness of the crystals in both cases is ca. 80 µm.

Crystallization of P6-C_{60}

c-P6-free (2.0 mg, 0.45 µmol) was dissolved in *o*-dichlorobenzene (0.15 mL) containing C_{60} (0.66 mg, 0.9 µmol, 2.0 equiv.) and placed in a small vial inside a larger vial filled with methanol, which was closed. After ten days at 20 °C, solution lost most of its green color, and crystals of **P6-C_{60}** were formed as dark green blocks.

Unsuccessful crystallization attempts

It is worth noting that the *o*-dichlorobenzene/methanol conditions were the only ones that provided single crystal diffraction-quality crystals. We tried combinations of methanol (and *n*-hexane) with other solvents, including benzene, toluene, chlorobenzene, 1,1,2,2-tetrachloroethane, chloroform, dichloromethane, tetrahydrofuran. We also found that **c-P6-free** co-crystallizes only with C_{60}, not C_{70}. The template-bound and template-free nanorings (with zinc cations present) were subjected to co-crystallization with C_{60} and C_{70} too, unsuccessfully. The only other successful crystallization attempt was with the template-bound nanoring and C_{60} that formed small hexagon-shaped crystals, but the diffraction was extremely weak (essentially two-dimensional diffraction patterns) and even unit cell determination was not possible, indicating lack of long-range order in the structure. Subjecting freshly obtained, smaller crystals to three-dimensional electron diffraction experiments (3D-ED, Rigaku XtaLab Synergy-ED) in cryo-conditions did not help either, indicating that the problem arises from the intrinsic nature of this material.

3. Measurement and refinement details

3.1. Crystal mounting

All crystals described in this work suffer from very quick solvent loss, which destroys their crystallinity. They should not be kept in the mother liquor too long, otherwise their diffraction power decreases significantly – it works best when they are fresh. Moreover, **P6-III** and **P6-IV** dissolve in all types of cryoprotecting oils that we have attempted (Fomblin YR-1800, Paratone, NVH oil – see Fig. S4); the slowest decomposition is observed in the thick Paratone and NVH oils, but crystals should be still handled within few seconds. Crystals of **P6-II** are relatively stable upon immersing in oil (Fig. S5). Decomposition of **P6-C_{60}** is slower than **P6-III** and **P6-IV** but faster than **P6-II**. Picking crystals at low temperature did not show any improvement in the data quality in comparison to very fast handling at room temperature. Mounting in a sealed capillary was similarly unsuccessful.

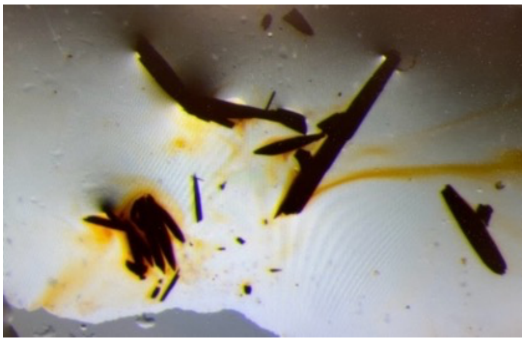

Figure S4. Crystals of **P6-III** and **P6-IV** dissolve shortly (<20 sec) after contact with Paratone oil. For scale, the width of the large plate is ca. 70 µm.

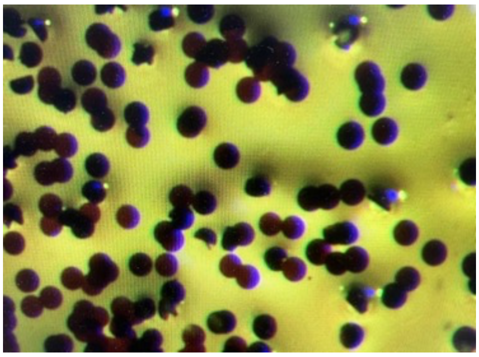

Figure S5. Crystals of **P6-II** remain intact two minutes after contact with Paratone oil. Average diameter of the crystals is 40 µm.

3.2. Measurement details

The crystals diffract very weakly (large unit cells, disorder, solvent loss) and as such, high-flux X-ray sources were crucial for the measurement. Unfortunately, beam damage was observed. Data collection using synchrotron radiation was attempted too (Diamond Light Source, I19 beamline), however, despite numerous attempts, we never managed to get better data than on our in-house high flux rotating anode source (Rigaku Synergy DW). Diffraction experiments took more than 30 full data collection attempts for each sample (with even more crystallization attempts for each one) and the best datasets were selected for structure solution and refinement.

Due to challenging nature of the crystals, the resolution of the datasets is between 0.95 and 1.13 Å (data were trimmed during processing), depending on a structure. Although the data quality and achieved resolution do not enable detailed analysis of structural parameters (bond lengths, angles), the models provide information about shape, flexibility and packing of the nanorings in the solid state; therefore, the obtained structures are fit for purpose, publishable and of great significance, considering the complexity of the samples.

3.3. Refinement details

The structures were solved with SHELXT (version 2018/2)[2] and refined by full-matrix least-squares procedures using SHELXTL (version 2019/2)[3] software package through the OLEX2 graphical interface.[4] All non-hydrogen atoms, including those in disordered parts, were refined anisotropically.

<u>Refinement of **P6-II, P6-III** and **P6-IV**</u>

Due to low data resolution, displacement parameters along the bonds involving non-metal atoms and their other components were restrained using global SIMU and RIGU restraints. *Tert*-butyl groups were modelled with aid of the residuals (RESI command), using appropriate similar distance restraints (SADI/DFIX), in some cases modelling them with PART-1 command when the group lays on symmetry element, adjusting occupancies appropriately. Disorder within the template and *tert*-butyl groups was modelled using standard techniques, splitting appropriate atoms into two PARTs and their occupancies were constrained at 0.5 to aid the refinement (free refinement using free variables indicated values close to 0.50, so the assumption is sensible). Appropriate restraints on the displacement parameters of disordered parts (SIMU) and distances (SADI/DFIX) were applied. The distances within pyridine motifs

were modelled using DFIX restraints on appropriate 1,2- and 1,3- distances, making them similar to these in a structure of a pyridine-coordinated porphyrin found in literature (CCDC: 912743).[5] Some phenyl rings within the template were constrained to be regular hexagons with bond length of 1.39 Å (AFIX 66 command), as attempts to model them using strong restraints did not bring satisfactory results. All restrained phenyl rings were restrained to be planar using FLAT command. Due to low data resolution, the hydrogen atoms in the *tert*-butyl groups were placed geometrically, using AFIX 33 option. The porphyrin and 1,3-butadiyne motifs were not subjected to additional geometric restraints to make sure they are not influenced by externally provided information which could impact the structural parameters such as degree of planarity and co-linearity. Solvent masking was employed to account for smeared electron density corresponding to disordered solvent molecules which could not be modelled; as such, parameters such as density of the crystal in the CIF are underestimated.

Refinement of **P6-C_{60}**

Due to low data resolution (no diffraction spots observed below 1.13 Å), displacement parameters along the bonds and their other components were restrained using global SIMU and RIGU restraints with default strength. *Tert*-butyl groups were modelled using standard restraints on appropriate 1,2- and 1,3 distances (SADI/DFIX). Ellipsoids in part of the aryl substituents appear as elongated, reflecting their vibrational motion; attempts to model them as disordered over two positions failed, and hence they were left after restraining them appropriately. Fullerene fragment in the asymmetric unit was modelled using a set of restraints available upon fitting C_{60} from the FragmentDB library,[6] cutting out atoms not constituting part of the asymmetric unit, and applying extra restraints on the displacement parameters (SIMU). Due to low data resolution, the hydrogen atoms in the *tert*-butyl groups were placed geometrically, using AFIX 33 option. The porphyrin and 1,3-butadiyne motifs were not a subject to additional geometric restraints to make sure they are not influenced by externally provided information which could impact the structural parameters such as degree of planarity and co-linearity. Solvent masking was employed to account for smeared electron density corresponding to disordered solvent molecules which could not be modelled; as such, parameters such as density of the crystal in the CIF are underestimated. Positions of hydrogen atoms in the porphyrin cores could not be located on a difference electron density map and as such, they were modelled as disordered over all four possible positions, with occupancy of 0.5 each.

Identification code	**P6-II**	**P6-III**	**P6-IV**	**P6-C_{60}**
CCDC number	2374893	2374895	2374896	2374894
Empirical formula	$C_{384}H_{348}N_{30}Zn_6$	$C_{384}H_{348}N_{30}Zn_6$	$C_{390}H_{352}Cl_2N_{30}Zn_6$	$C_{240}H_{172}Cl_8N_{12}$
Formula weight	5775.12	5775.12	5922.11	3507.49
Temperature/K	100.00(10)	100.00(10)	100.00(10)	100.01(10)
Crystal system	trigonal	trigonal	triclinic	monoclinic
Space group	*P*-3m1	*P*-3m1	*P*-1	*I*2/m
a/Å	49.3794(3)	49.74500(17)	20.6135(2)	20.1388(3)
b/Å	49.3794(3)	49.74500(17)	38.7108(3)	53.4956(7)
c/Å	28.6911(5)	55.2247(4)	39.1085(4)	31.8306(4)
α/°	90	90	81.8780(10)	90
β/°	90	90	80.2100(10)	106.7920(10)
γ/°	120	120	74.8970(10)	90
Volume/Å^3	60585.6(13)	118348.4(12)	29535.9(5)	32830.1(8)
Z	4	8	2	4
ρ_{calc} / g/cm^3	0.633	0.648	0.666	0.71
μ/mm^{-1}	0.513	0.525	0.614	0.898
F(000)	12168	24336	6232	7328
Crystal size/mm^3	0.05 × 0.05 × 0.005	0.28 × 0.13 × 0.12	0.22 × 0.11 × 0.04	0.24 × 0.14 × 0.12
Radiation	Cu Kα (λ = 1.54184)	Cu Kα (λ = 1.54184)	Cu Kα (λ = 1.54184)	Cu Kα (λ = 1.54184)
2Θ range for data collection/°	3.08 to 89.466	3.552 to 100.87	3.474 to 109.042	7.994 to 86.264
Reflections collected	535747	1089116	584195	234517
Independent reflections	16792	43210	72418	12095
R_{int}	0.086	0.0896	0.0657	0.0573
R_{sigma}	0.0328	0.023	0.0361	0.0190]
Data/restraints/parameters	16792/5279/1534	43210/10741/2680	72418/21316/4166	12095/4278/1171
GooF on F^2	1.067	1.028	1.03	1.016
Final R_1/wR_2	0.1041 / 0.3224	0.1474 / 0.4219	0.1815 / 0.5064	0.1395 / 0.4051
Final R_1/wR_2 [all data]	0.1320 / 0.3558	0.1759 / 0.4794	0.2045 / 0.5353	0.1626 / 0.4512
Largest diff. peak/hole / e Å^{-3}	0.96/-0.44	1.31/-0.69	1.07/-0.73	0.58/-0.52

Table S1. Single crystal X-ray diffraction measurement and refinement details.

Due to substantial size of the CIF files, they had to be processed manually by the Cambridge Crystallographic Data Centre (CCDC) deposition team. As a result of the CCDC software limitations, the *checkcif* reports did not go through their usual processing and those that can be accessed *via* the Access Structures service appear as corrupted. The .cif and .hkl files had to be separated due to their large size but they can be accessed normally *via* the Access Structures service and the *checkcif* tests can be performed on them as usual.

We cite all checkcif A and B level alerts and our responses to them below.

P6-II

Alert level A

```
THETM01_ALERT_3_A  The value of sine(theta_max)/wavelength is less than 0.550
            Calculated sin(theta_max)/wavelength =    0.4565
```

Author Response: The crystals were a subject of more than 30 data collections, including attempts using synchrotron radtiation. The best dataset was selected for structure solution and refinement and although the obtained resolution is ca. 1.1 Angstrom, the data provided a useful model showing the flexibility and packing of the molecules in the solid state.

Alert level B

```
PLAT084_ALERT_3_B High wR2 Value (i.e. > 0.25) ...................       0.36 Report
```

Author Response: Related to the poor quality of crystals and their poor diffraction power (see the answer to the level A alert).

P6-III

Alert level A

```
THETM01_ALERT_3_A  The value of sine(theta_max)/wavelength is less than 0.550
            Calculated sin(theta_max)/wavelength =    0.5000
```

Author Response: The crystals were a subject of more than 30 data collections, including attempts using synchrotron radtiation. The best dataset was selected for structure solution and refinement and although the obtained resolution is ca. 1.0 Angstrom, the data provided a useful model showing the flexibility and packing of the molecules in the solid state. The crystals suffer from solvent loss immediately after removing them from mother liquor, dissolve in cryoprotecting oils and are susceptible to the beam damage.

```
PLAT084_ALERT_3_A High wR2 Value (i.e. > 0.25) ...................       0.48 Report
```

Author Response: Related to the poor data quality, see the response to the level A alert.

Alert level B

```
PLAT973_ALERT_2_B Check Calcd Positive Resid. Density on       Zn4       1.92 eA-3
```

Author Response: Most likely caused by absorption and poor data quality; attempts to correct for absorption using methods with face indexing did not improve it.

```
PLAT973_ALERT_2_B Check Calcd Positive Resid. Density on       Zn1       1.84 eA-3
```

Author Response: Most likely caused by absorption and poor data quality; attempts to correct for absorption using methods with face indexing did not improve it.

```
PLAT973_ALERT_2_B Check Calcd Positive Resid. Density on       Zn5       1.78 eA-3
```

Author Response: Most likely caused by absorption and poor data quality; attempts to correct for absorption using methods with face indexing did not improve it.

```
PLAT973_ALERT_2_B Check Calcd Positive Resid. Density on       Zn3       1.63 eA-3
```

Author Response: Most likely caused by absorption and poor data quality; attempts to correct for absorption using methods with face indexing did not improve it.

P6-IV

Alert level A

```
THETM01_ALERT_3_A  The value of sine(theta_max)/wavelength is less than 0.550
            Calculated sin(theta_max)/wavelength =    0.5282
```

Author Response: Crystals diffracted poorly, more than 30 data collections were done, including experiments using synchrotron radiation and the best dataset was selected for solution and refinement. Despite poor quality and low resolution (no reflections beyond 0.95 Angstroem) the model provides useful information regarding geometry, flexibility and packing of these nanorings in the solid state.

```
PLAT084_ALERT_3_A High wR2 Value (i.e. > 0.25) ...................       0.54 Report
```

Author Response: Consequence of poor data quality (see the answer to the other level A alert).

Alert level B

```
PLAT082_ALERT_2_B High R1 Value ..................................       0.18 Report
```

Author Response: Consequence of poor crystal quality (see response to the level A alert).

```
PLAT341_ALERT_3_B Low Bond Precision on  C-C Bonds ...............    0.01762 Ang.
```

Author Response: Consequence of poor crystal quality (see response to the level A alert).

P6-C_{60}

Alert level A

```
THETM01_ALERT_3_A  The value of sine(theta_max)/wavelength is less than 0.550
            Calculated sin(theta_max)/wavelength =    0.4434
```

Author Response: The crystals were a subject of numerous data collections, including attempts using synchrotron radtiation. The best dataset was selected for structure solution and refinement and although the dataser was measured up to 1.13 Angstrom due to lack of reflections beyond that resolution, the data provided a useful model showing the flexibility and packing of the molecules in the solid state.

Alert level B

```
PLAT084_ALERT_3_B High wR2 Value (i.e. > 0.25) ...................       0.45 Report
```

Author Response: Related to poor quality of the crystals (see the answer to the level A alert).

```
PLAT242_ALERT_2_B Low    'MainMol' Ueq as Compared to Neighbors of        C11 Check
```

Author Response: Related to poor quality of the crystals and disorder (see the answer to the level A alert).

```
PLAT242_ALERT_2_B Low    'MainMol' Ueq as Compared to Neighbors of        C39 Check
```

Author Response: Related to poor quality of the crystals and disorder (see the answer to the level A alert).

```
PLAT340_ALERT_3_B Low Bond Precision on  C-C Bonds ...............    0.01496 Ang.
```

Author Response: Related to poor quality of the crystals (see the answer to the level A alert).

```
PLAT910_ALERT_3_B Missing # of FCF Reflection(s) Below Theta(Min).         13 Note
                1  1  0,    0  2  0,    1  3  0,    0  4  0,   -1  0  1,    1  0  1,
                0  1  1,   -1  2  1,    1  2  1,    0  3  1,    0  0  2,   -1  1  2,
                0  2  2,
```

Author Response: Reflections most likely hidden behind a beamstop.

4. Crystal structure analysis

4.1. Displacement ellipsoid plots and packing

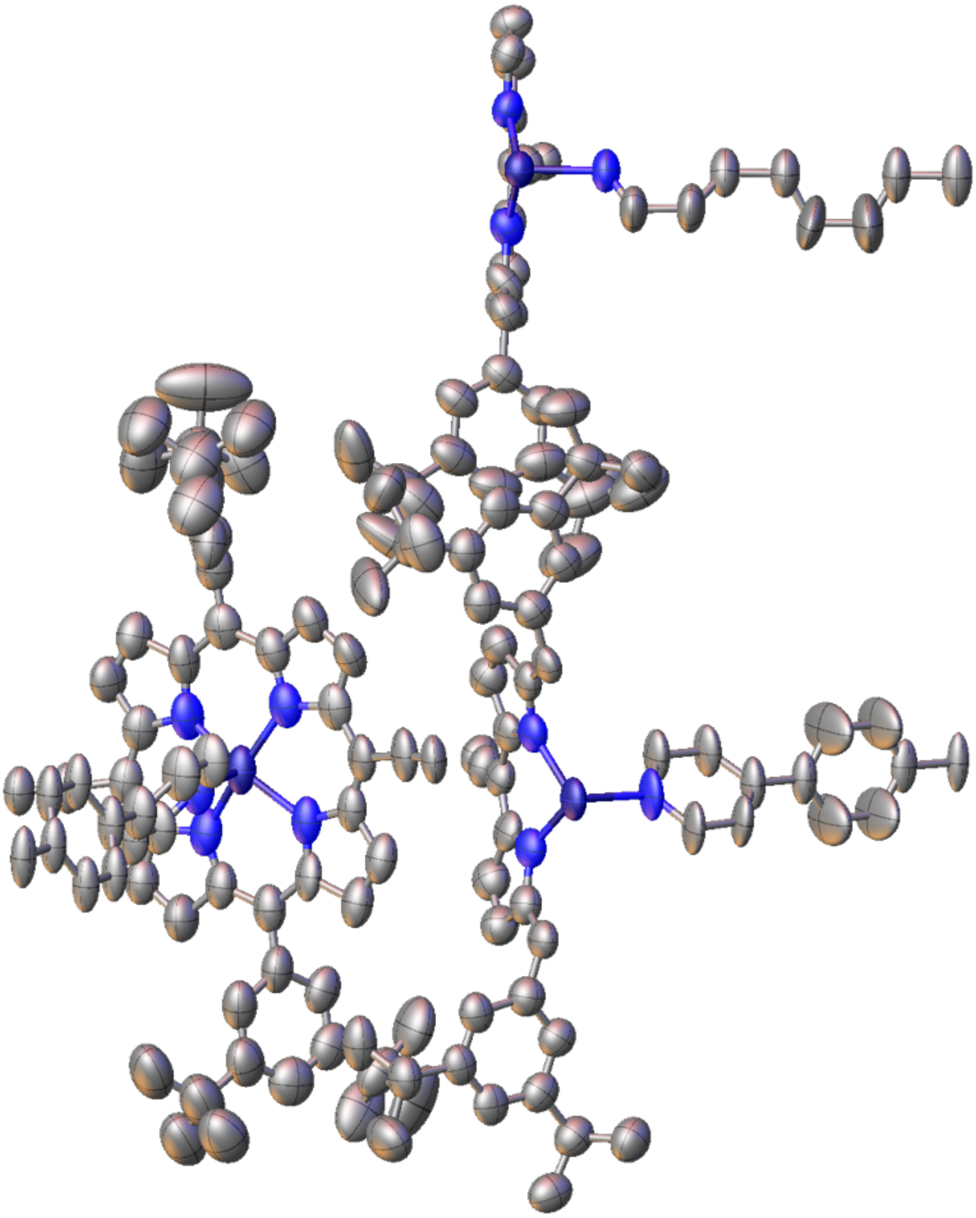

Figure S6. Displacement ellipsoid plot (50% probability level) for the asymmetric unit of **P6-II**. Hydrogen atoms and minor disorder components are not shown for clarity.

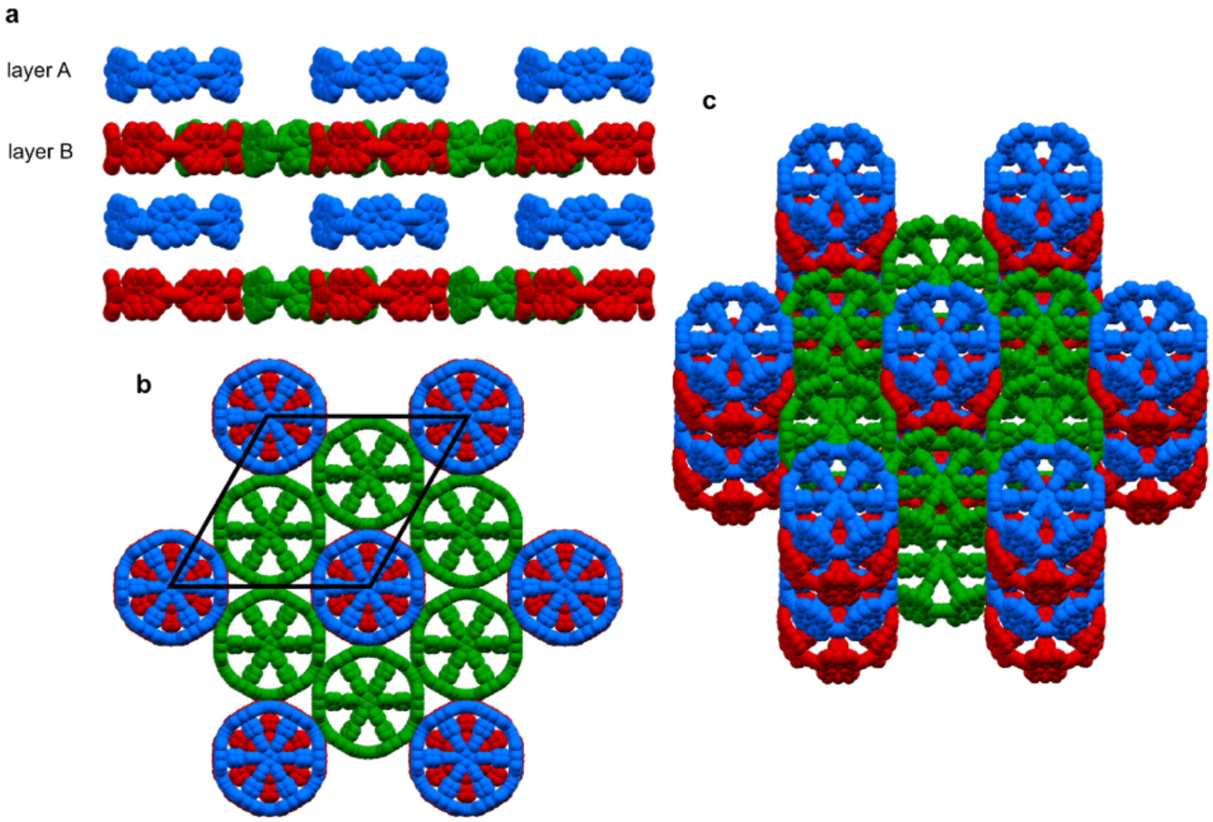

Figure S7. Schematic representation of packing in **P6-II**.

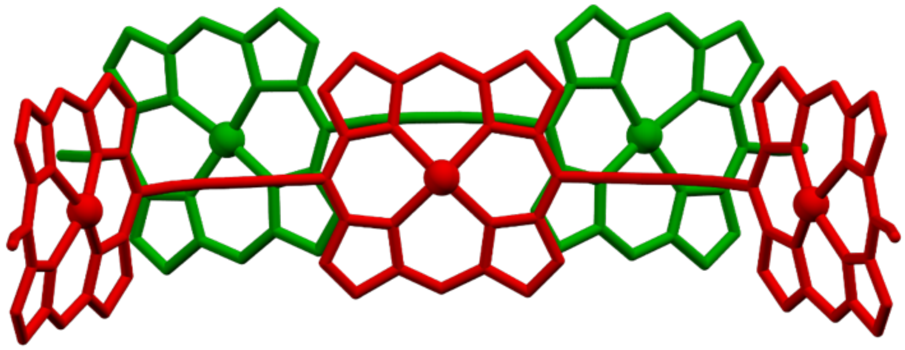

Figure S8. Schematic representation of packing in **P6-II**, view on the intermolecular butadiyne-porphyrin contact in the layer B (referred to Fig. S7).

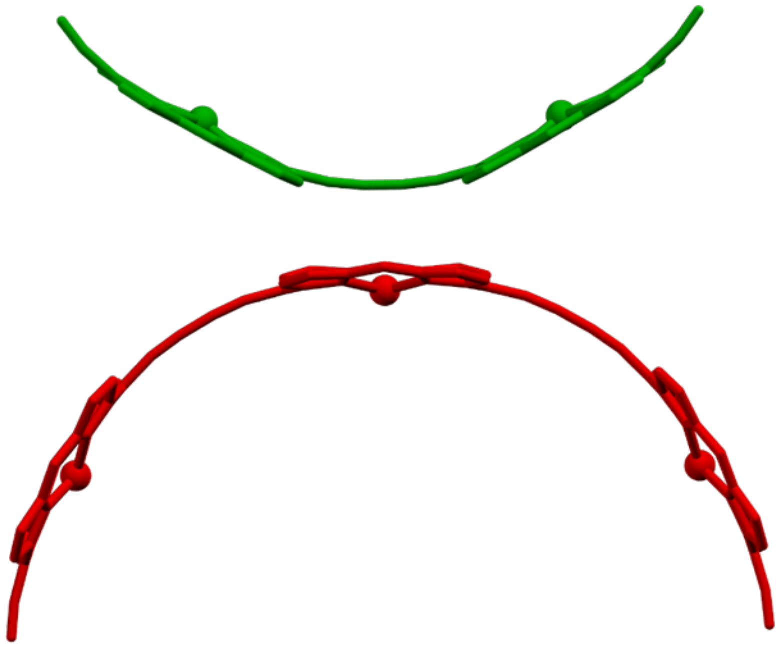

Figure S9. Schematic representation of packing in **P6-II**, view on the intermolecular butadiyne-porphyrin contact in the layer B (referred to Fig. S7).

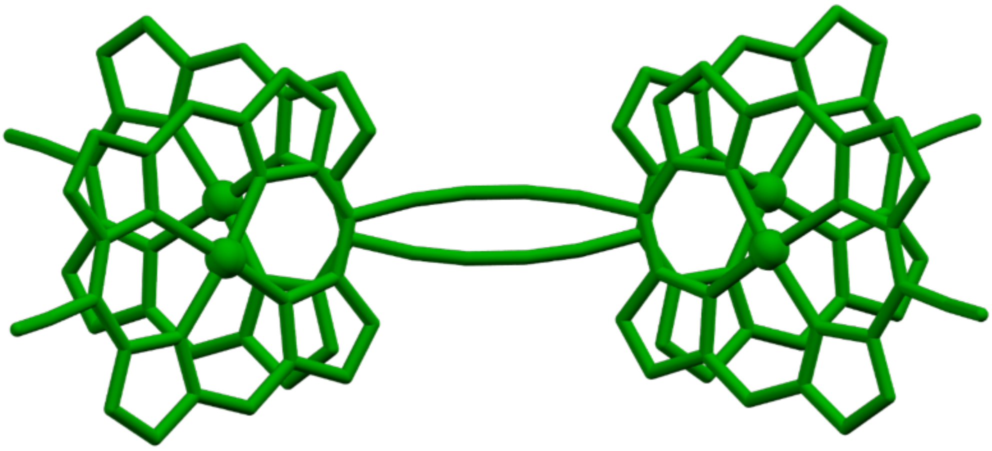

Figure S10. Schematic representation of packing in **P6-II**, view on the intermolecular butadiyne-butadiyne contact in the layer B (referred to Fig. S7).

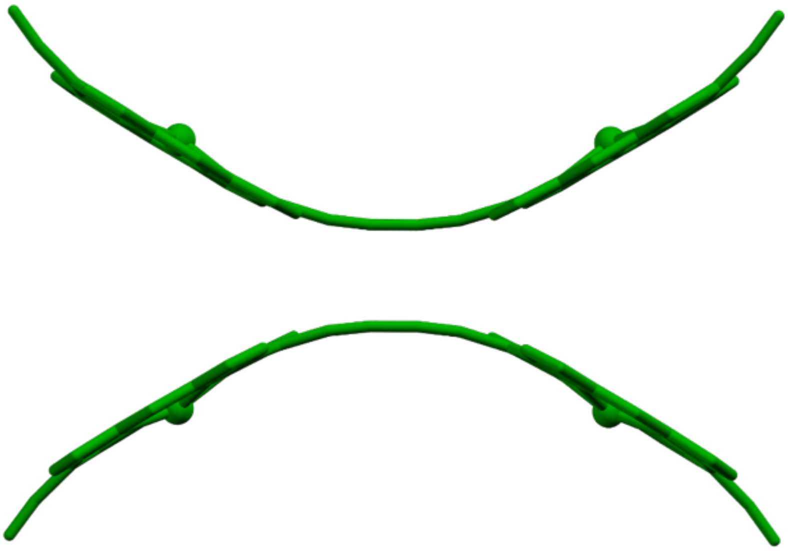

Figure S11. Schematic representation of packing in **P6-II**, view on the intermolecular butadiyne-butadiyne contact in the layer B (referred to Fig. S7).

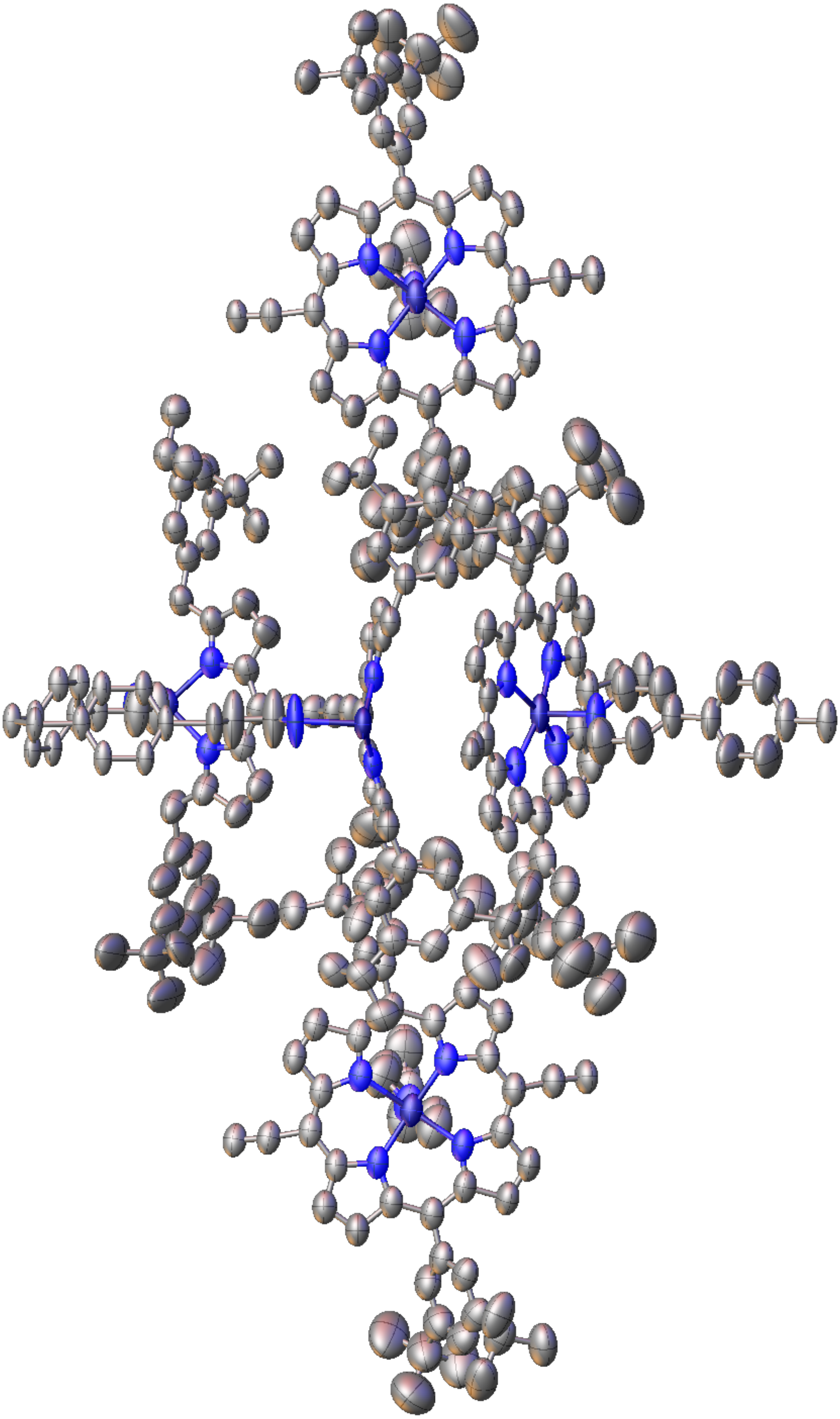

Figure S12. Displacement ellipsoid plot (50% probability level) for the asymmetric unit of **P6-III**. Hydrogen atoms and minor disorder components are not shown for clarity.

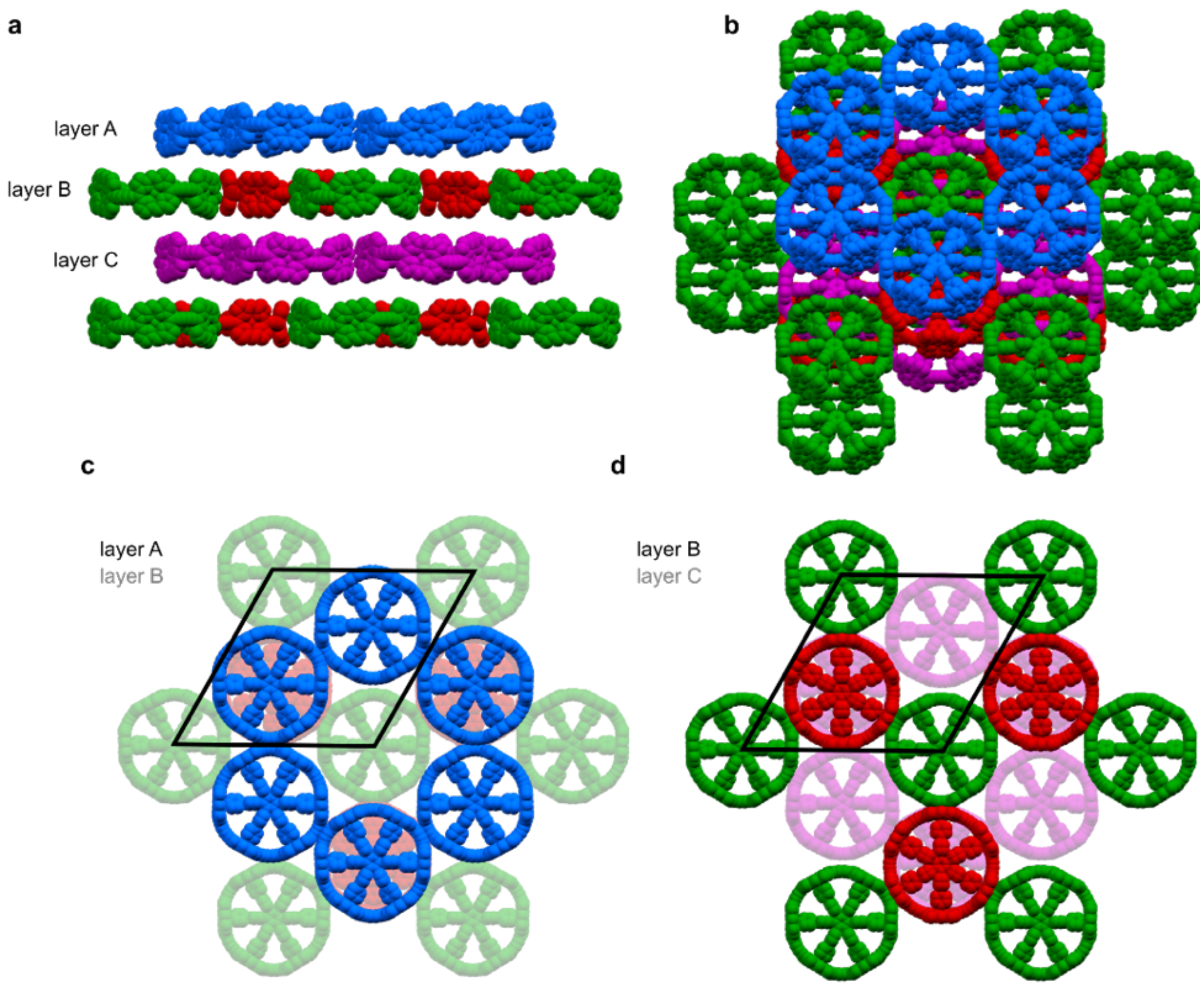

Figure S13. Schematic representation of packing in **P6-III**.

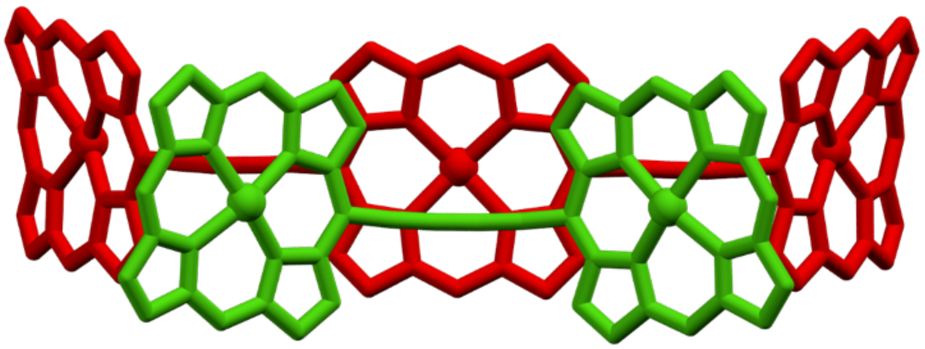

Figure S14. Schematic representation of packing in **P6-III**, view on the intermolecular butadiyne-porphyrin contact in the layer B (referred to Fig. S13).

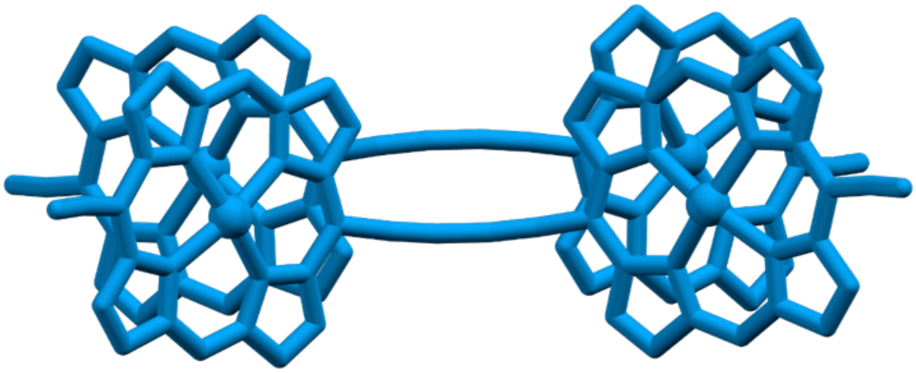

Figure S15. Schematic representation of packing in **P6-III**, view on the intermolecular butadiyne-butadiyne contact in the layer A (referred to Fig. S13).

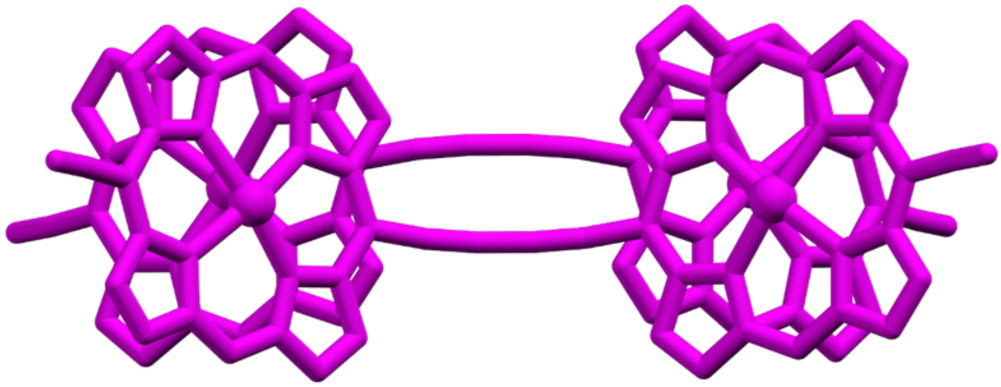

Figure S16. Schematic representation of packing in **P6-III**, view on the intermolecular butadiyne-butadiyne contact in the layer C (referred to Fig. S13).

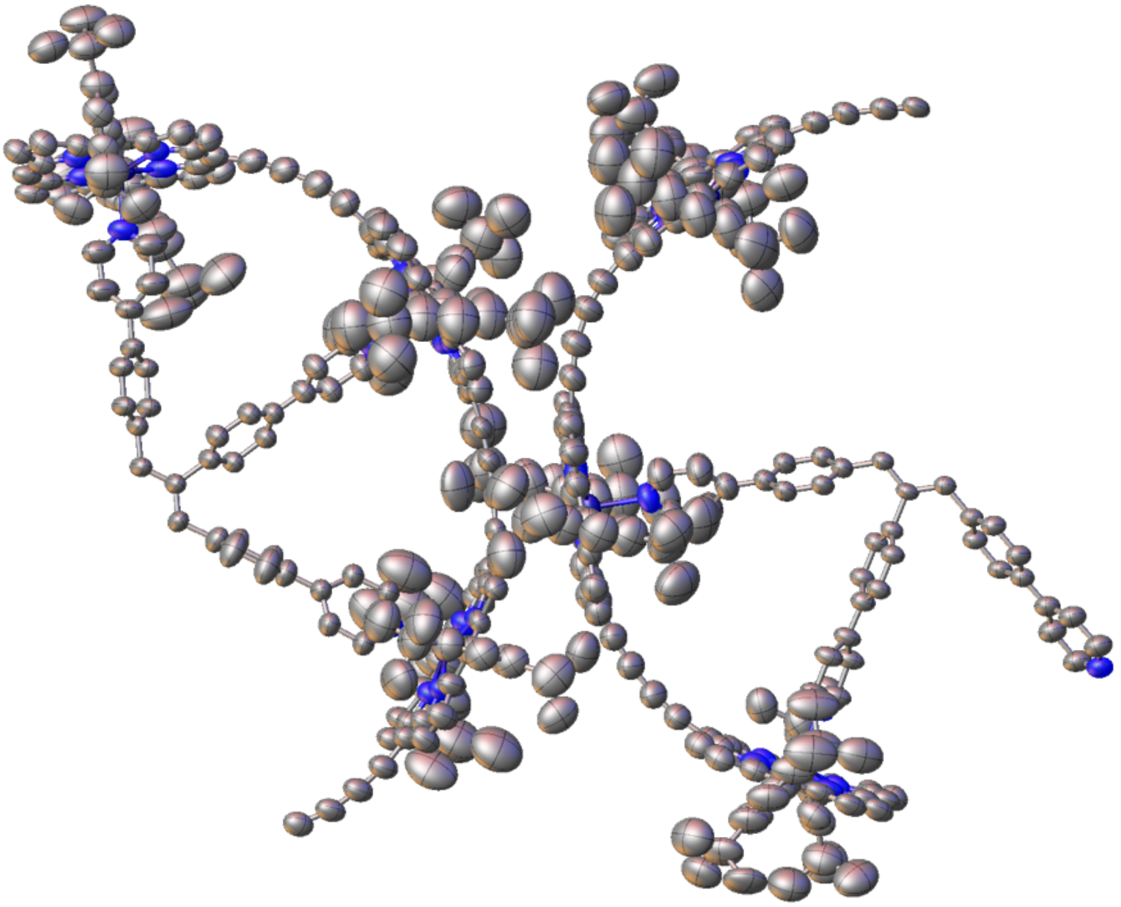

Figure S17. Displacement ellipsoid plot (50% probability level) for the asymmetric unit of **P6-IV**. Hydrogen atoms, modelled solvent molecule (*o*-dichlorobenzene) and minor disorder components are not shown for clarity.

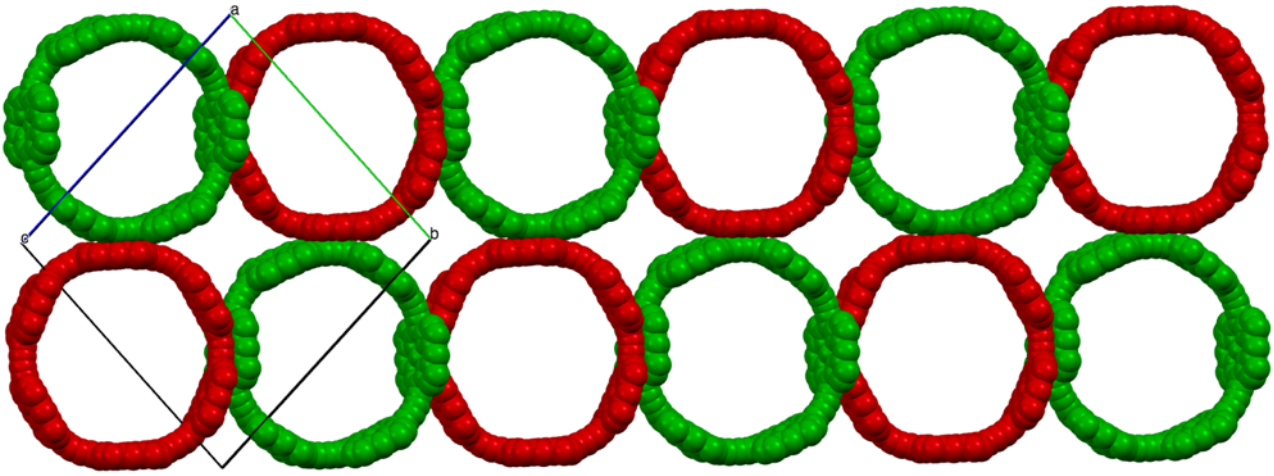

Figure S18. Packing in **P6-IV**.

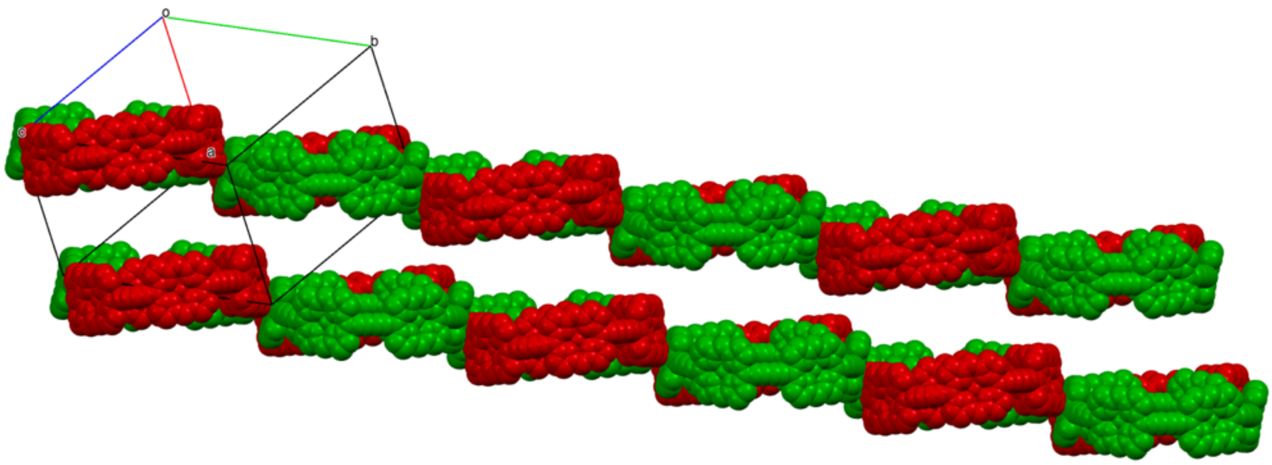

Figure S19. Packing in **P6-IV**.

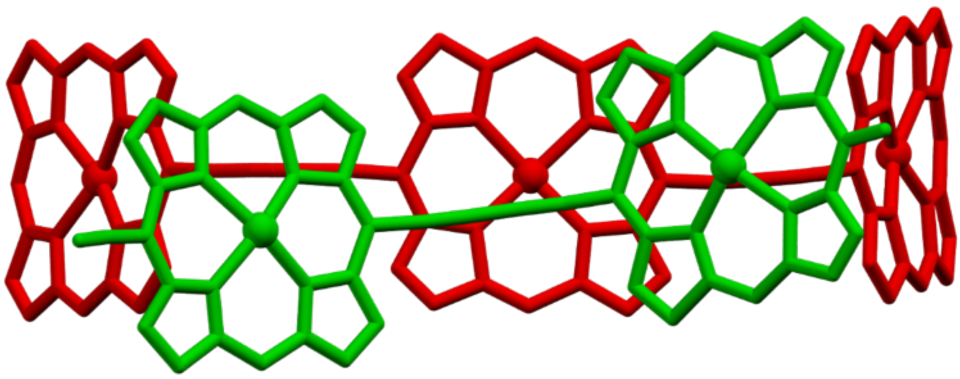

Figure S20. Schematic representation of packing in **P6-IV**, view on the intermolecular butadiyne-porphyrin contact (referred to Fig. S18-S19).

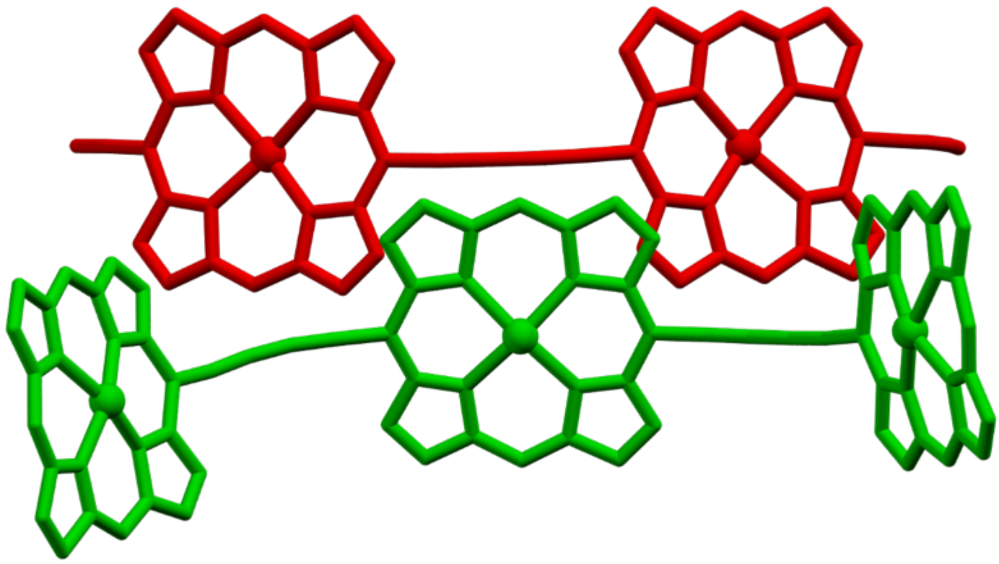

Figure S21. Schematic representation of packing in **P6-IV**, view on the intermolecular butadiyne-porphyrin contact (referred to Fig. S18-S19).

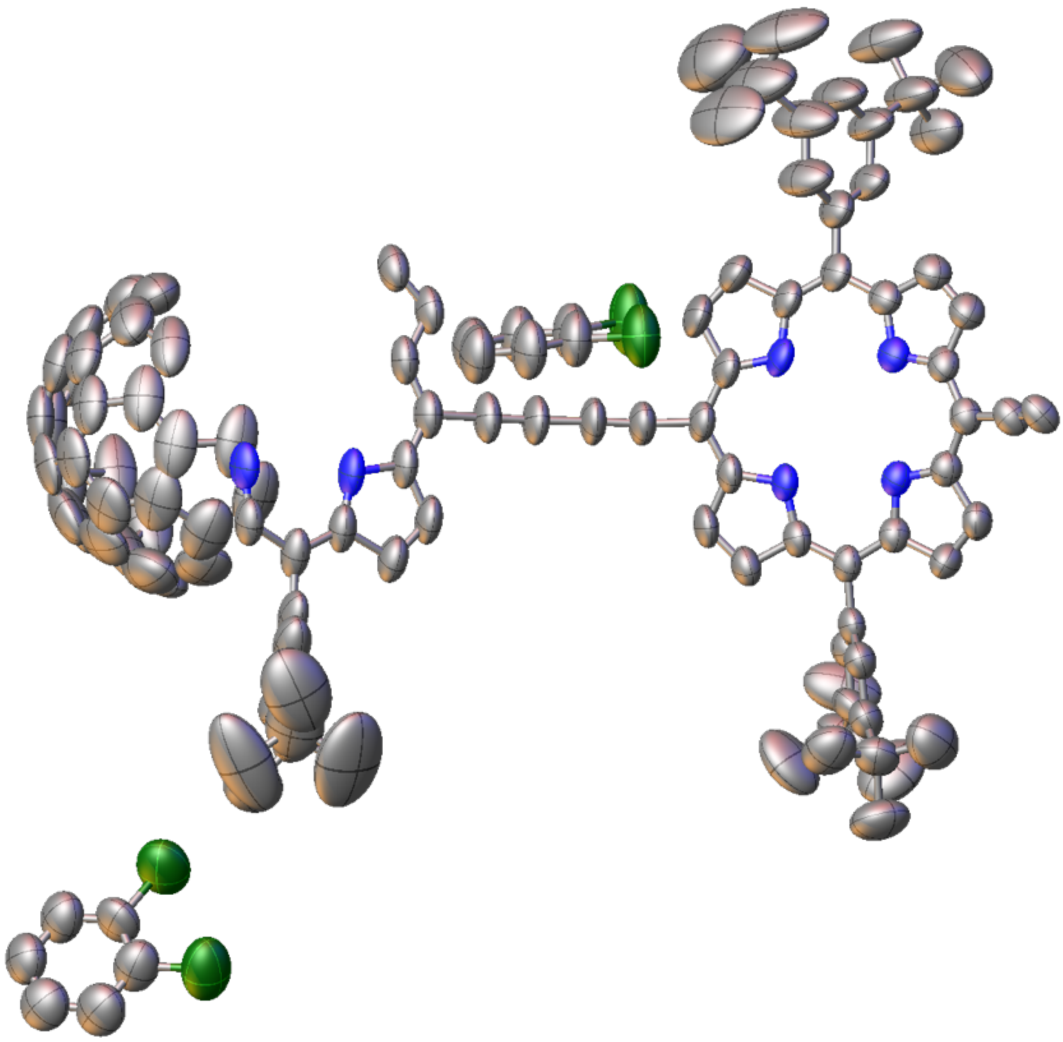

Figure S22. Displacement ellipsoid plot (50% probability level) for the asymmetric unit of **P6-C_{60}**. Hydrogen atoms are not shown for clarity.

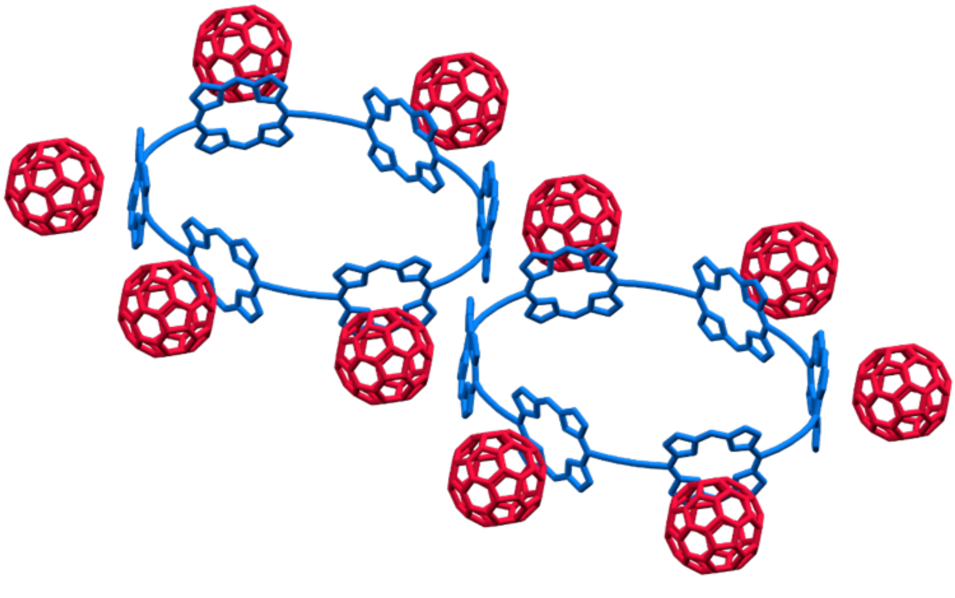

Figure S23. Schematic representation of packing in **P6-C_{60}**, emphasizing the intermolecular butadiyne-butadiyne contacts.

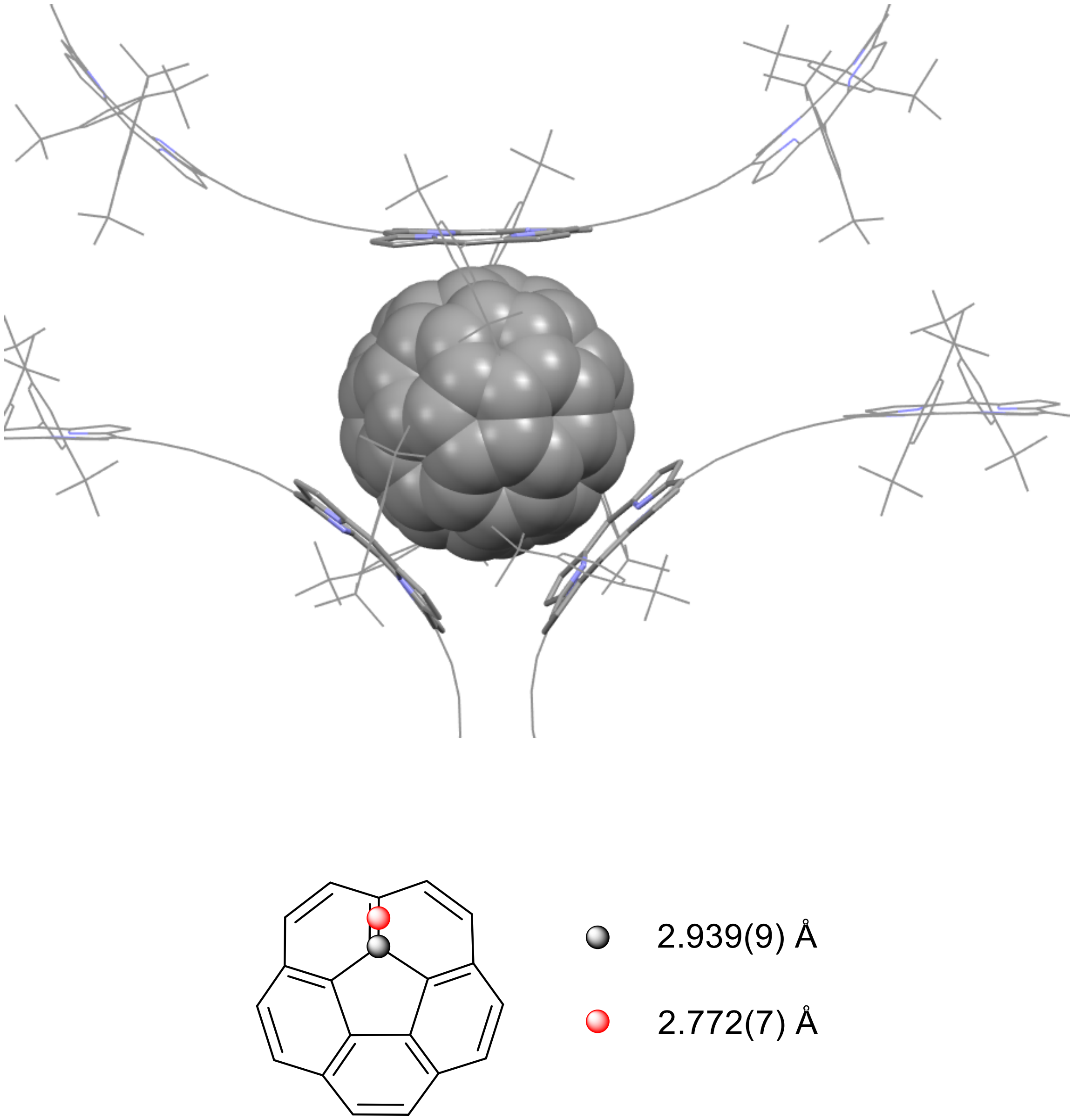

Figure S24. Stacking of fullerene C_{60} to porphyrin walls with indicated shortest fullerene atom/two-atom centroid – porphyrin centroid distances (calculated with Olex2 software[4]).

Solvent accessible void calculations were performed using Mercury software[7] using a spherical probe radius of 1.2 Å, using structures with fully removed solvent molecules.

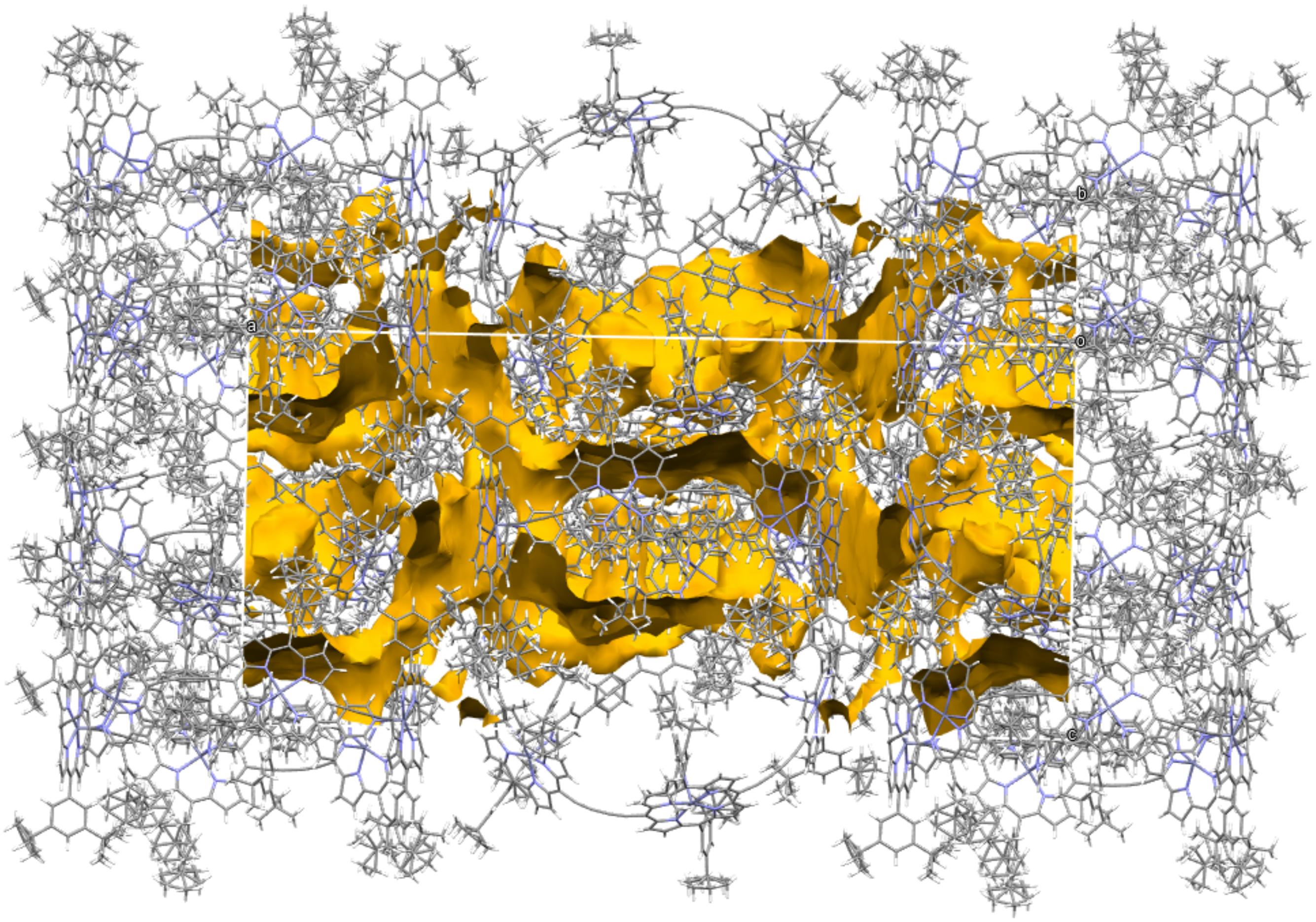

Figure S25. Visualization of solvent-accessible voids in **P6-I**. Voids constitute 13.2% of the unit cell volume.

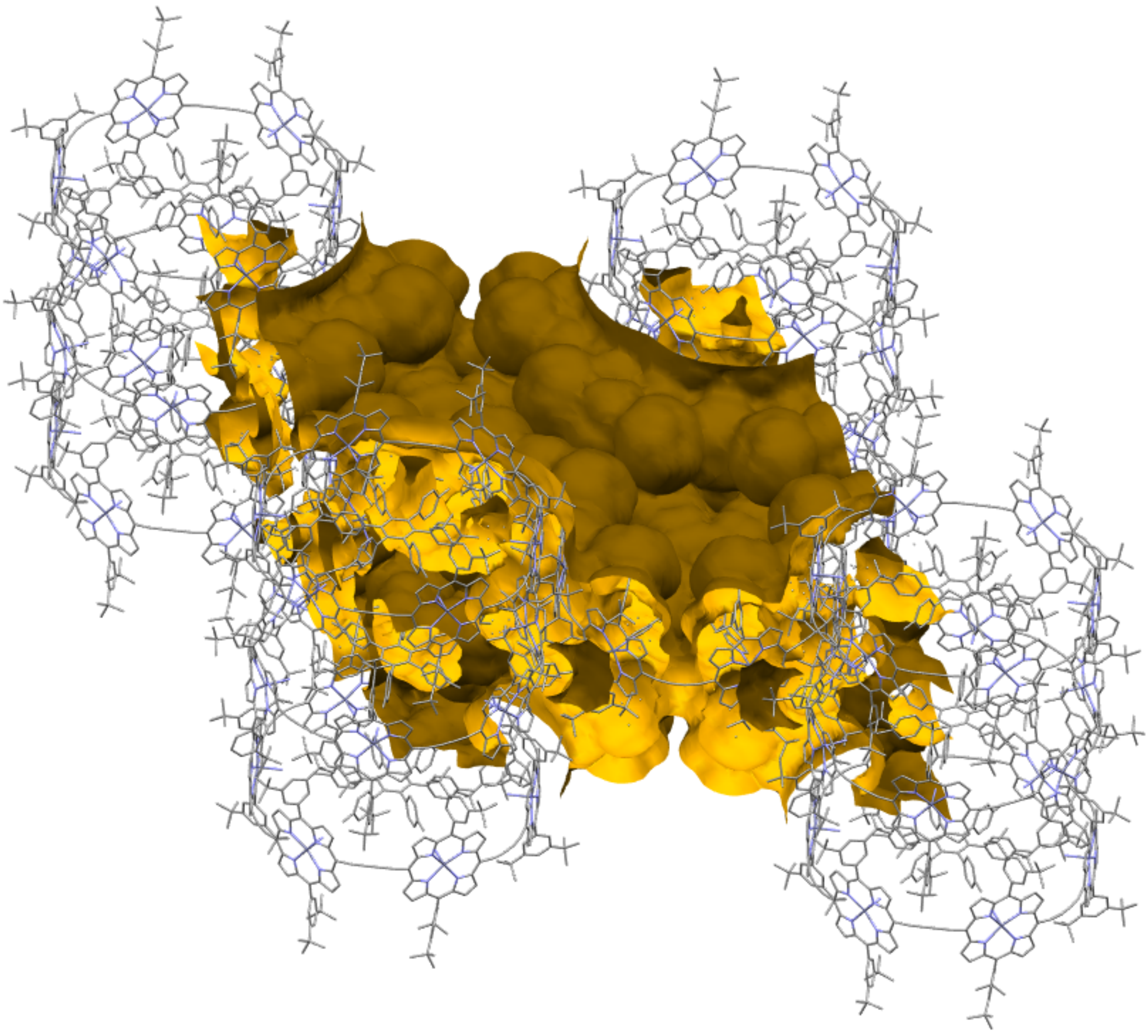

Figure S26. Visualization of solvent-accessible voids in **P6-II**. Voids constitute 33.0% of the unit cell volume.

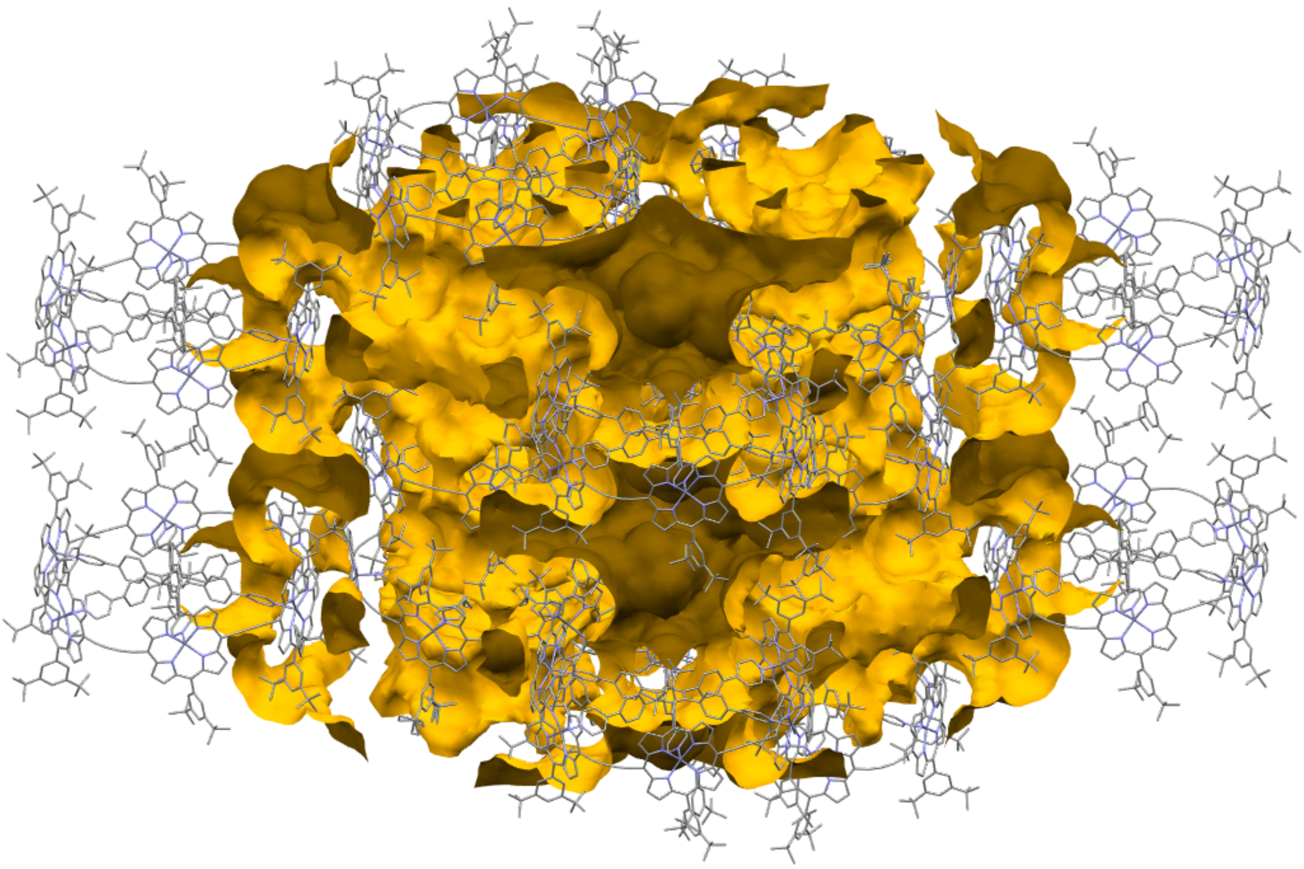

Figure S27. Visualization of solvent-accessible voids in **P6-III**. Voids constitute 32.7% of the unit cell volume.

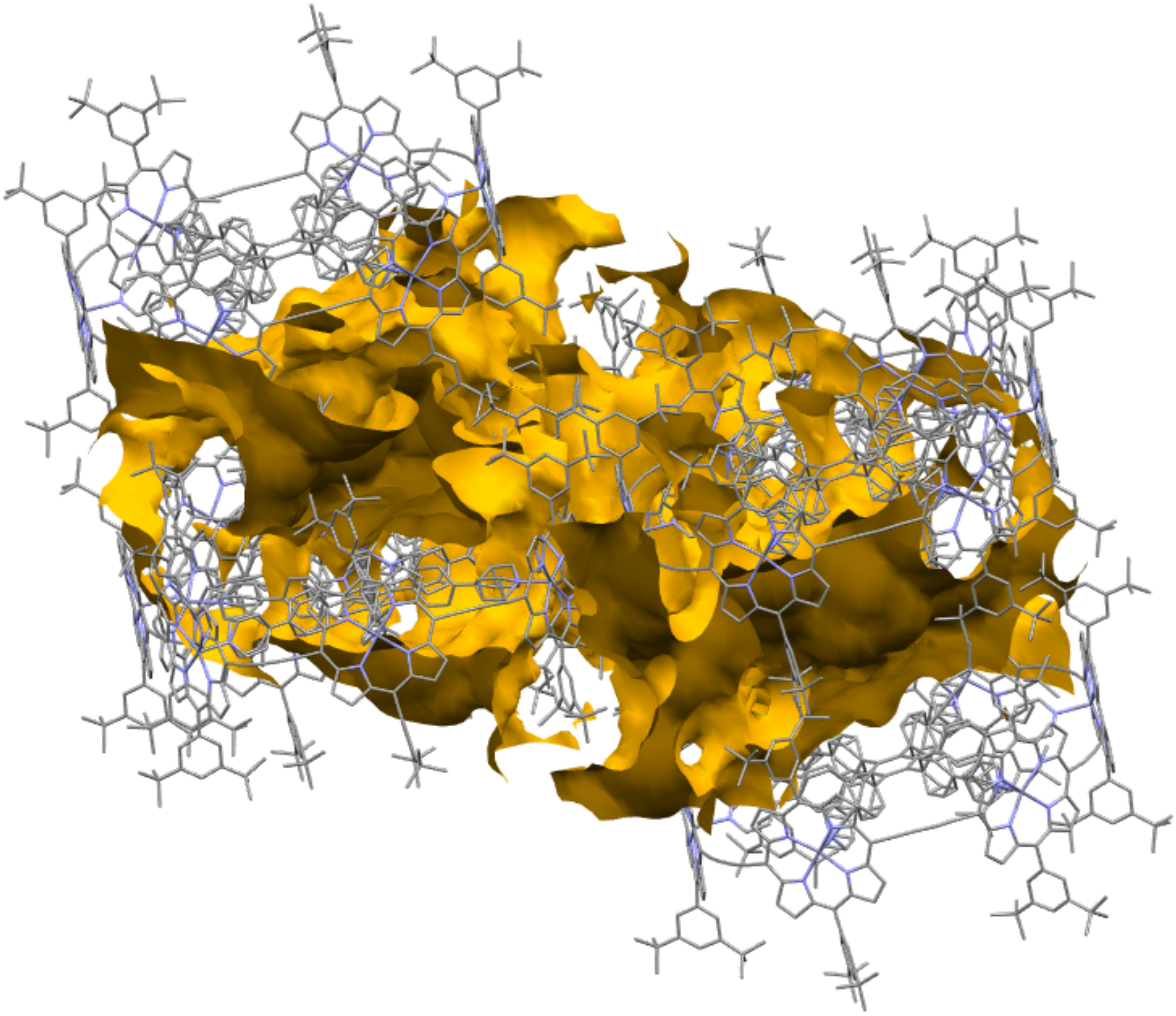

Figure S28. Visualization of solvent-accessible voids in **P6-IV**. Voids constitute 28.4% of the unit cell volume.

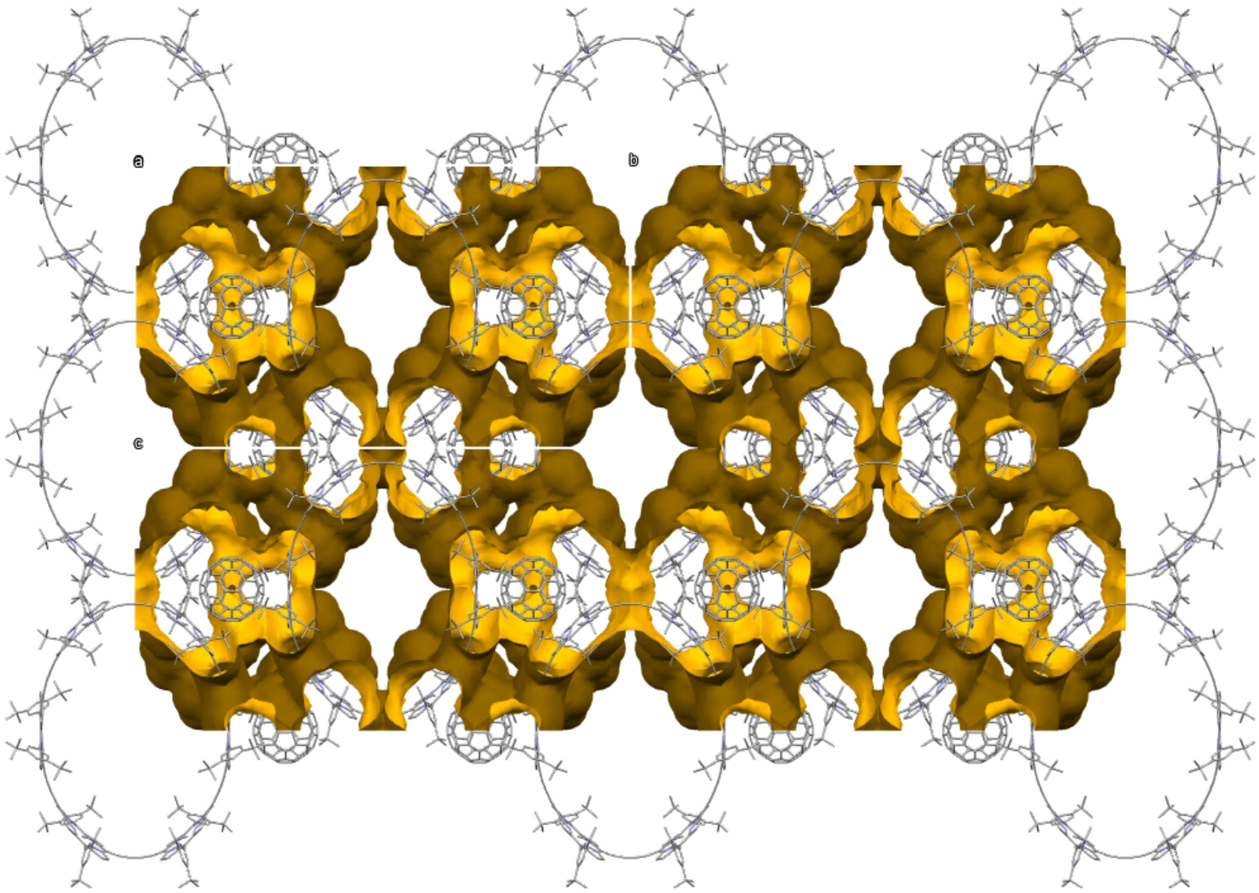

Figure S29. Visualization of solvent-accessible voids in **P6-C_{60}**. Voids constitute 40.3% of the unit cell volume.

4.2. Geometrical parameters

The geometrical parameters obtained after crystal structure refinement are given below. For clarity and as a representative example, Fig. S30 shows geometry of the two types of nanorings in the structure of **P6-II** and visualized planes of Zn atoms (green) and porphyrin planes (violet). It is clearly visible that the porphyrins in the conformer with bendy butadiyne linkers are relatively planar, whereas in the conformer with linkers approximately in the Zn plane, porphyrins are bent towards inside.

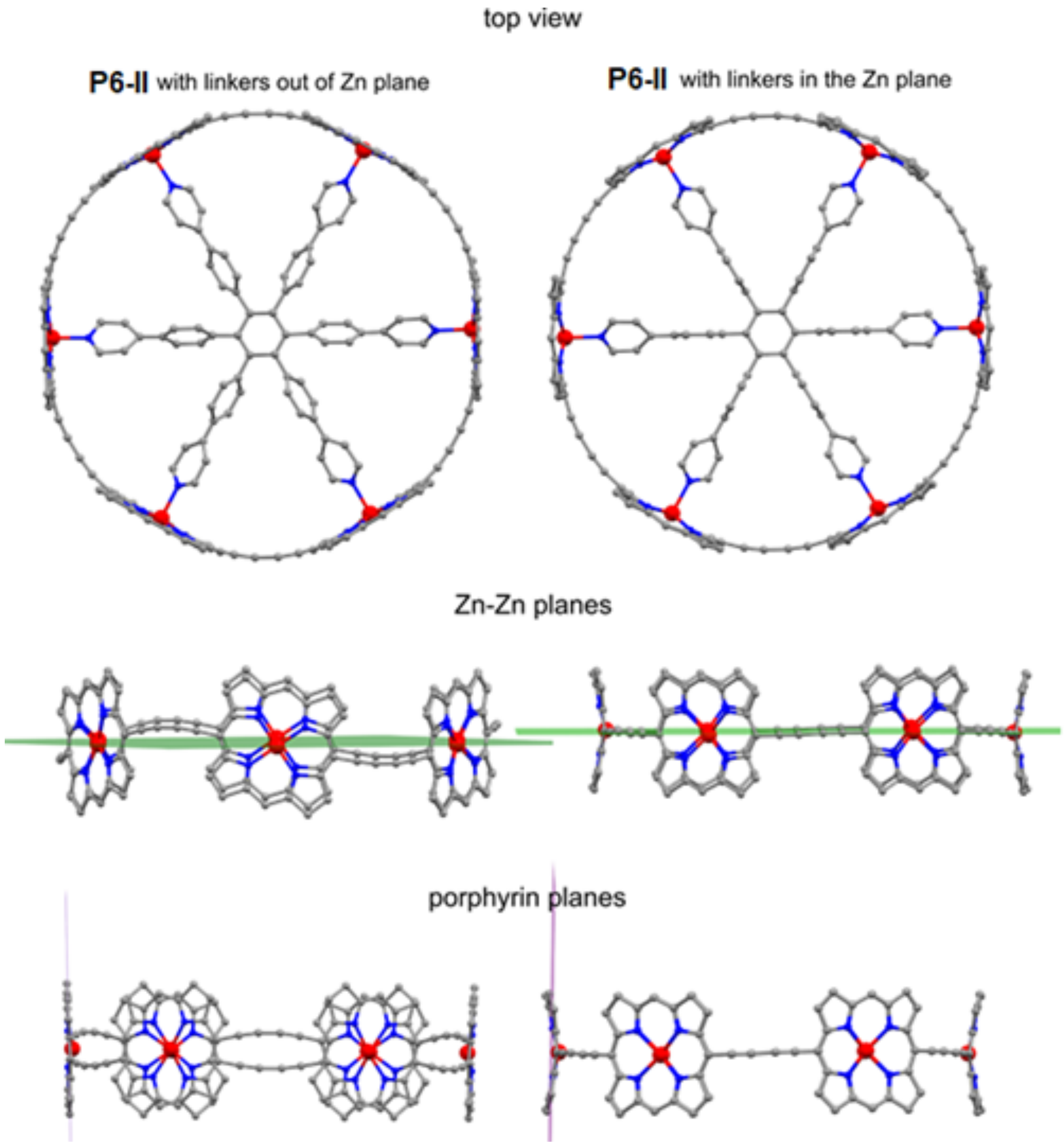

Figure S30. Molecular structure and defined Zn and porphyrin planes for two conformers in **P6-II**.

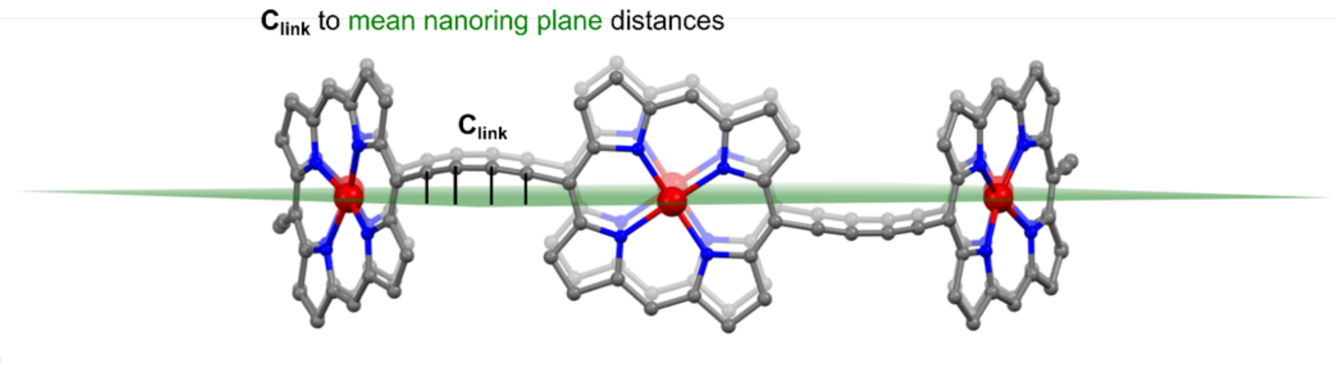

Figure S31. Depiction of the distance between carbon atoms in the butadiyne likers (***C_{link}***) and Zn atoms plane given in Table S2.

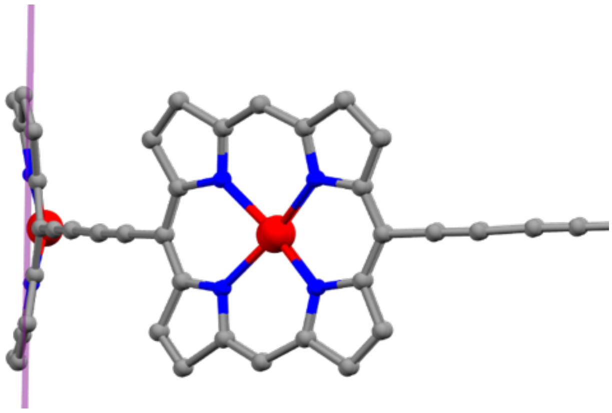

Figure S32. Depiction of the mean porphyrin plane (violet) as an illustration for values given in Table S2.

**P6-I**[1]						
Zn plane RMSD	0.096					
porphyrin plane RMSD	0.139	0.086	0.067			
Zn-porphyrin plane dist	0.2859(17)	0.261(2)	0.173(2)			
C_{link} to Zn plane dist	0.716(10)	0.884(10)	0.907(10)	0.882(9)		
	0.777(10)	0.884(10)	0.881(9)	0.836(9)		
	0.690(9)	0.858(9)	0.911(9)	0.906(10)		
**P6-II**						
Zn plane RMSD	0	0.088	0			
porphyrin plane RMSD	0.204	0.046	0.061			
Zn-porphyrin plane dist	0.439(2)	0.221(2)	0.230(2)			
C_{link} to Zn plane dist	0.086(9)	0.037(9)	0.899(9)	0.983(9)	0.894(9)	1.023(9)
	0.746(9)	0.855(9)				
**P6-III**						
Zn plane RMSD	0.162	0	0	0		
porphyrin plane RMSD	0.226	0.177	0.08	0.059	0.078	
Zn-porphyrin plane dist	0.498(2)	0.431(3)	0.2428(15)	0.270(2)	0.265(2)	
C_{link} to Zn plane dist	0.148(9)	0.041(10)	0.078(10)	0.117(10)		
	0.761(11)	0.78(1)	0.855(8)	0.910(11)		
	0.613(11)	0.632(9)	0.701(8)	0.670(11)		
	1.035(10)	0.926(10)	1.056(8)	0.933(9)		
**P6-IV**						
Zn plane RMSD	0.052	0.123				
porphyrin plane RMSD	0.086	0.073	0.121	0.165	0.137	0.07
Zn-porphyrin plane dist	0.354(3)	0.318(3)	0.3526(19)	0.301(3)	0.397(3)	0.341(2)
C_{link} to Zn plane dist	0.334(11)	0.327(13)	0.282(13)	0.244(13)		
	0.273(17)	0.261(15)	0.243(15)	0.204(13)		
shaded = Zn4, Zn5, Zn6	0.365(13)	0.358(13)	0.363(13)	0.292(13)		
blank = Zn1, Zn2, Zn3	0.486(11)	0.465(11)	0.332(11)	0.095(11)		
	0.337(10)	0.297(10)	0.284(11)	0.254(11)		
	0.493(10)	0.438(14)	0.329(14)	0.253(13)		
**P6-C_{60}**						
mean plane RMSD	0.024					
porphyrin plane RMSD	0.155	0.073				
C_{link} to mean plane dist	0.133(11)	0.180(11)	0.016(10)	0.008(12)	0.003(11)	0.011(11)

Table S2. Structural parameters determined for the crystal structures. The colors were used to separate parameters from different nanoring molecules and correspond to the colors of the nanorings throughout figures in Section 4.1. Zn-porphyrin plane distance is the distance of the zinc atom from the 24-atom porphyrin plane.

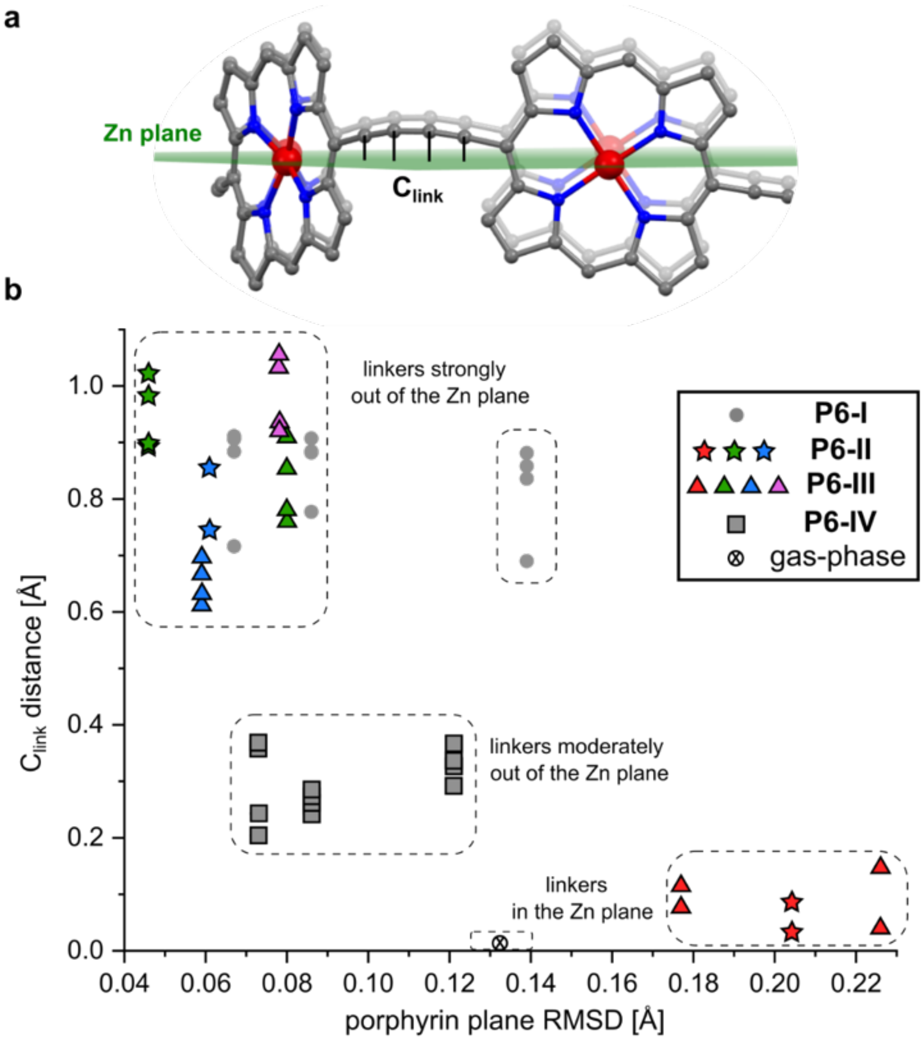

Figure S33. A diagram showing dependence of the porphyrin plane RMSD on the C_{link} distance. Colors for data points in case of P6-II and P6-III corresponds to colors of nanorings in Fig. S7 and S13. DFT = BLYP35 6-31G*.[8]

5. NMR and MS spectra

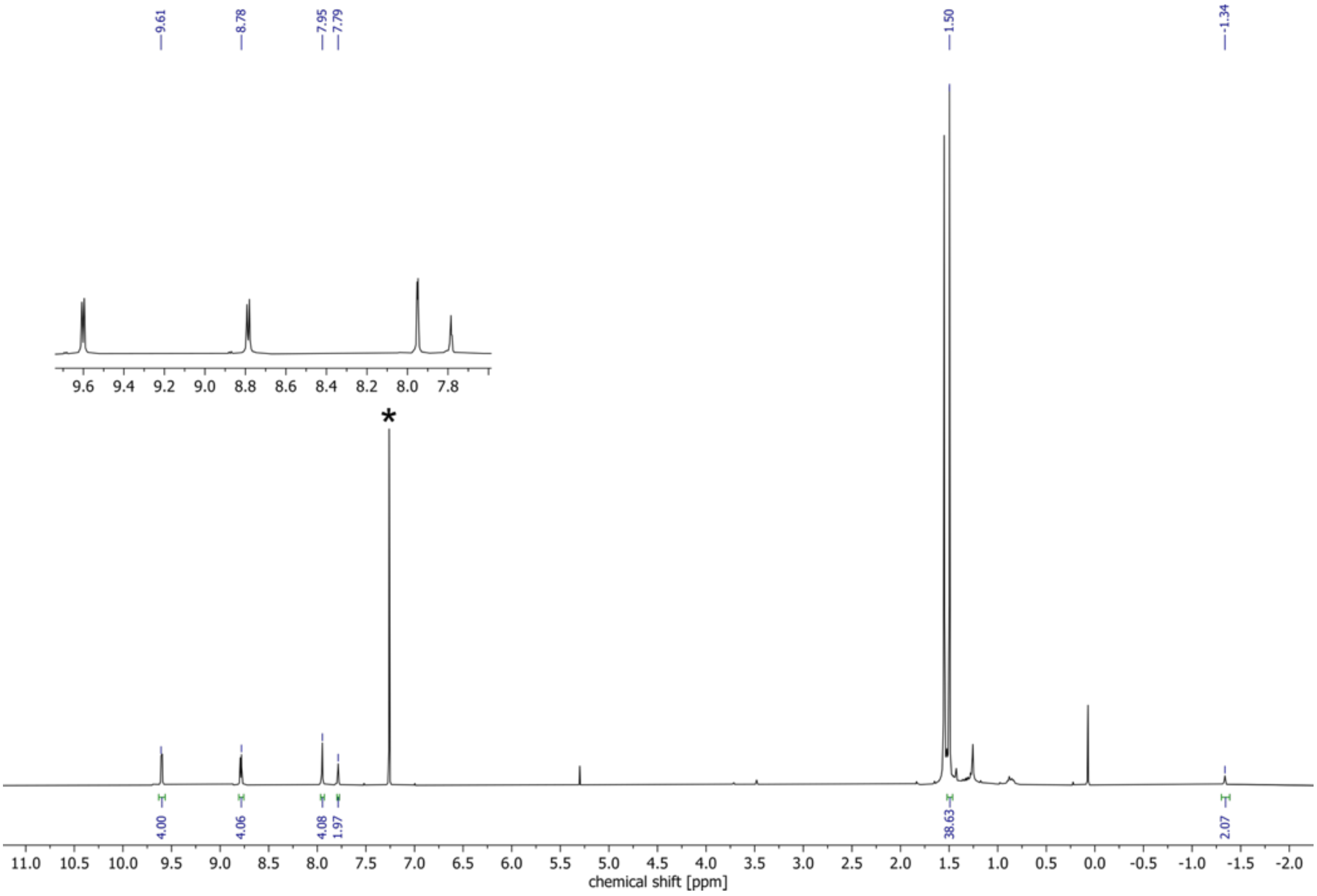

Figure S34. ^{1}H NMR spectrum of **cP6-free**, $CDCl_3$, 400 MHz, 298 K. Asterisk indicates residual solvent signal ($CHCl_3$).

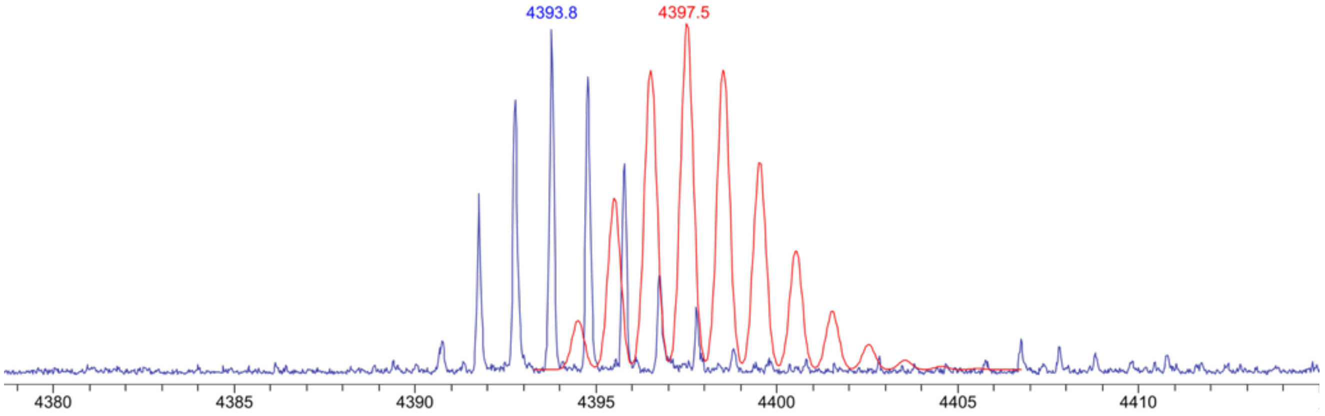

Figure S35. Fragment of recorded (blue) and simulated (red) MALDI mass spectra of **cP6-free**, DCTB matrix. The accuracy of the mass is low, but the simulated and observed isotopic patterns agree well (it is related to the large mass recorded and general performance of our instrument). Attempts to utilize ESI and APCI ionization were unsuccessful.

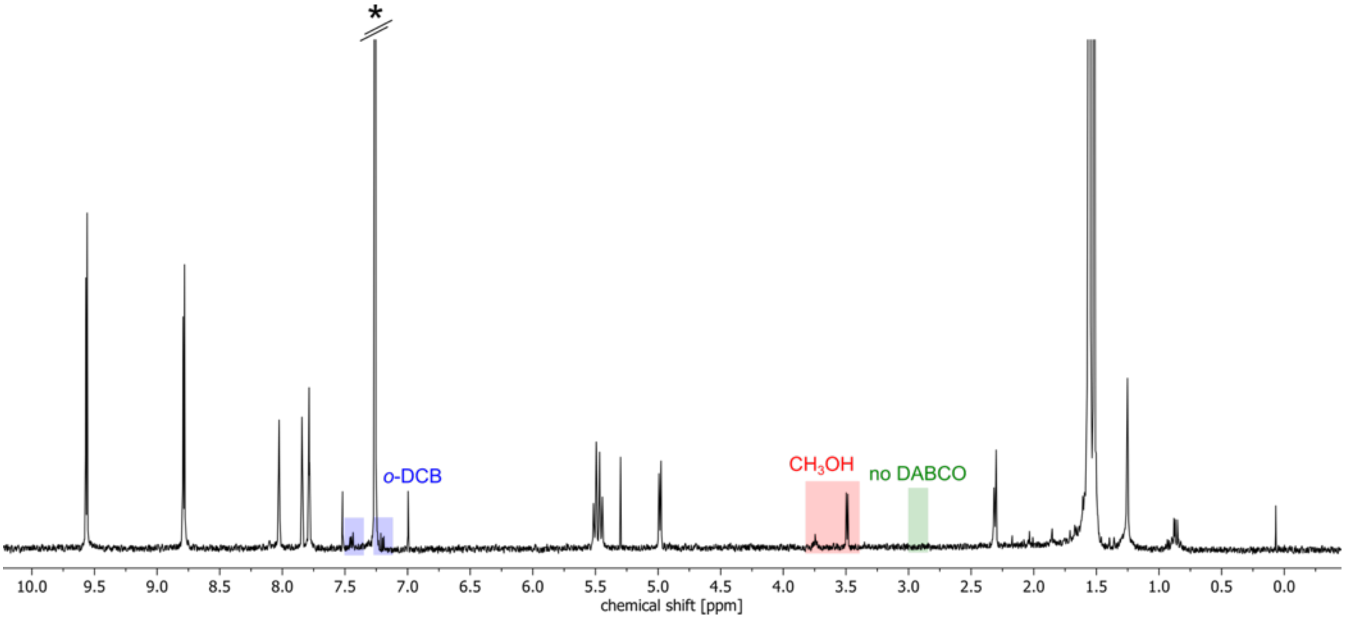

Figure S36. ^{1}H NMR spectrum of the dissolved crystals of **P6-II**. $CDCl_3$, 400 MHz, 298 K. Solvent and amorphous suspension were decanted, and crystals were dried in vacuum for 2 min and then redissolved in deuterated chloroform. The spectrum suggests lack of DABCO in the crystal structure and shows presence of *o*-dichlorobenzene and methanol.

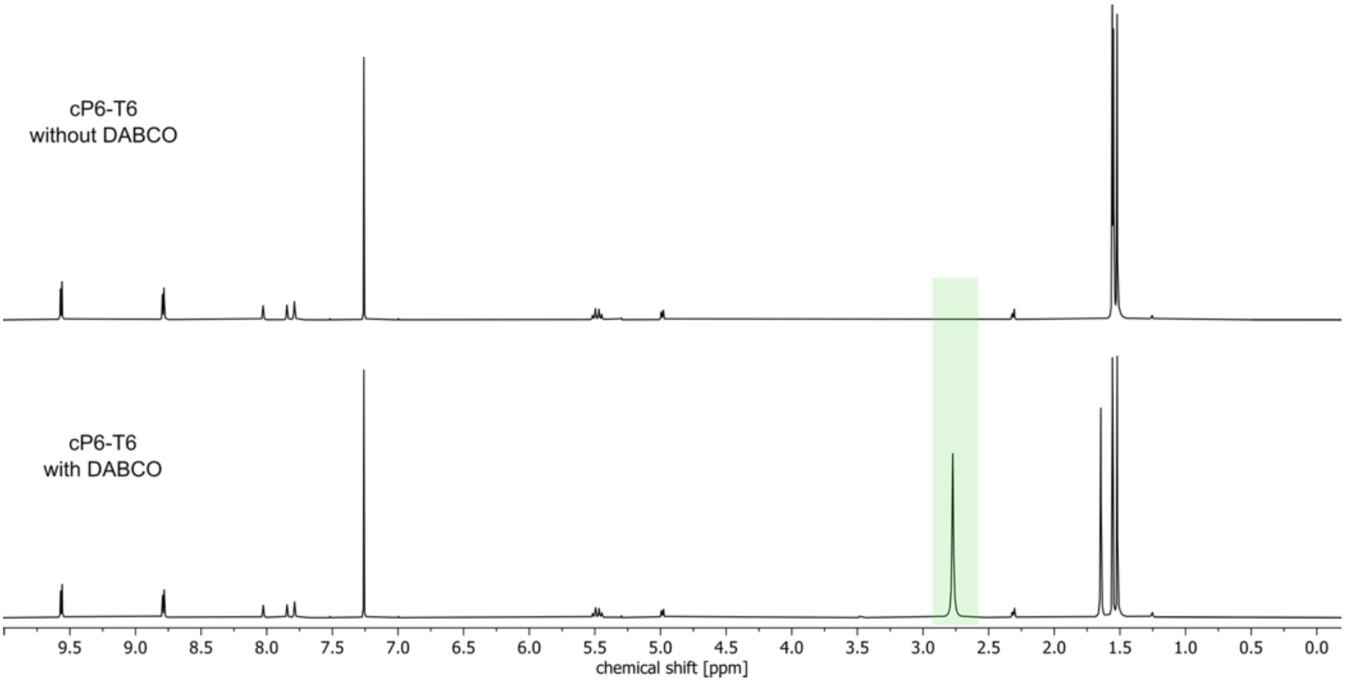

Figure S37. Control experiment – addition of DABCO (excess) to the solution of **cP6-T6**, $CDCl_3$, 400 MHz, 298 K. DABCO signal is marked in green.

6. Theoretical calculations

6.1. **c-P6·T6**

Calculation of the geometry of the template-bound **c-P6·T6** is problematic due to the very large size of the molecule and presence of the template with freely rotating phenyl and pyridine moieties and therefore, there are many points on the potential energy surface. The reported structures in the literature that were subsequently used to calculate properties, for example ^{1}H NMR spectra and ring currents, are different with respect to the planarity of porphyrins, shape of the template and bendiness of the butadiyne linkers.[8-9] Therefore, in such cases single crystal X-ray crystallography provides an extremely useful information about the shape the molecule adopts in the solid state which is ambiguous when considering only calculated models. Of course, an additional problem is that in the solid state, there are many more interactions that impact molecular shape than during gas-phase calculations on a single molecule. Nonetheless, it is useful to know which one of the geometries is closest to the energetic minimum.

We performed calculations to find out which one of the observed and/or calculated geometries is lowest in energy in the gas phase.

We started by taking two geometries from the crystal structure of **P6-II**, one with butadiyne linkers in the mean Zn plane, and the second one with wavy linkers, both from the layer B shown in Fig. S7 (red and green, respectively). The aryl *meso* substituents were replaced with hydrogen atoms to simplify the calculations. Geometry optimization on both structures was performed using Grimme's xbt method (GFN2-xtb, version 6.5.0) due to large size regime of the system and as a compromise between accuracy and the use of the computational resources.[10] The nanoring with 'straight' butadiyne linkers remained straight and the nanoring with bendy linker remained bendy in the optimized structure (with aryl groups deleted and replaced with H atoms). The energy difference is 54.4 kJ/mol in favor of the geometry with 'straight' linkers. The existence of a local minimum was verified by frequency calculation (--hess command). Negative frequencies were not observed.

Next, we performed two independent conformational searches using CREST software (version 2.10.2)[11] staring from both xtb-optimized geometries: bendy and straight. The two molecular dynamics runs converged on an identical geometry, which exhibits bending in the butadiyne linkers, but different to that observed in the crystal structure: the periodicity of waving of the butadiyne linkers pointing upwards is twice the one observed in the solid state (Fig. S38-S39). Moreover, it is 2.0 kJ/mol lower in energy than the xtb geometry with the 'straight' butadiyne linkers. A diagram comparing energies of the obtained structures is shown in Fig. S40.

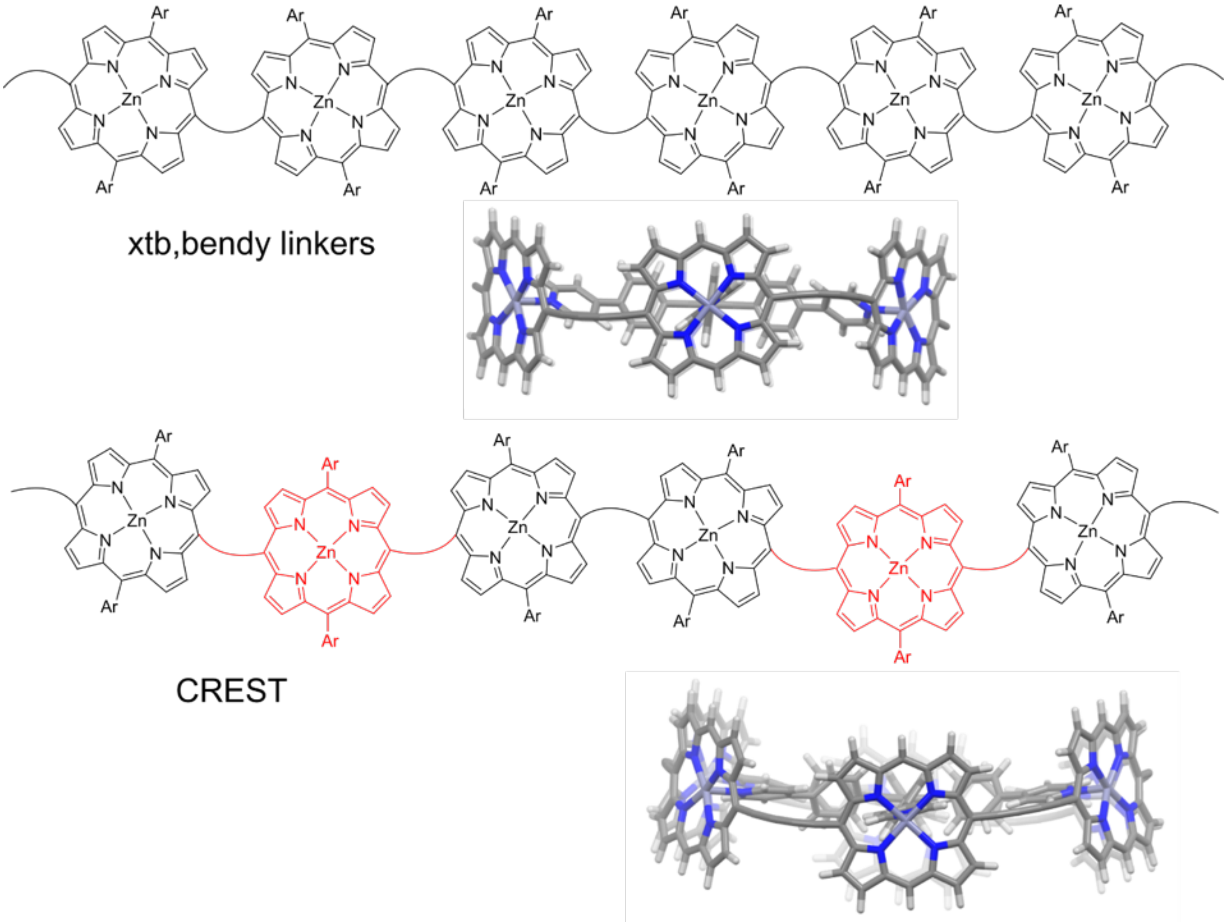

Figure S38. Comparison of the propagation of the bend in the linkers in the xtb-optimized structure based on the crystal structure and the result of the CREST computational search. The red porphyrins in the drawing have linkers pointing in the same direction.

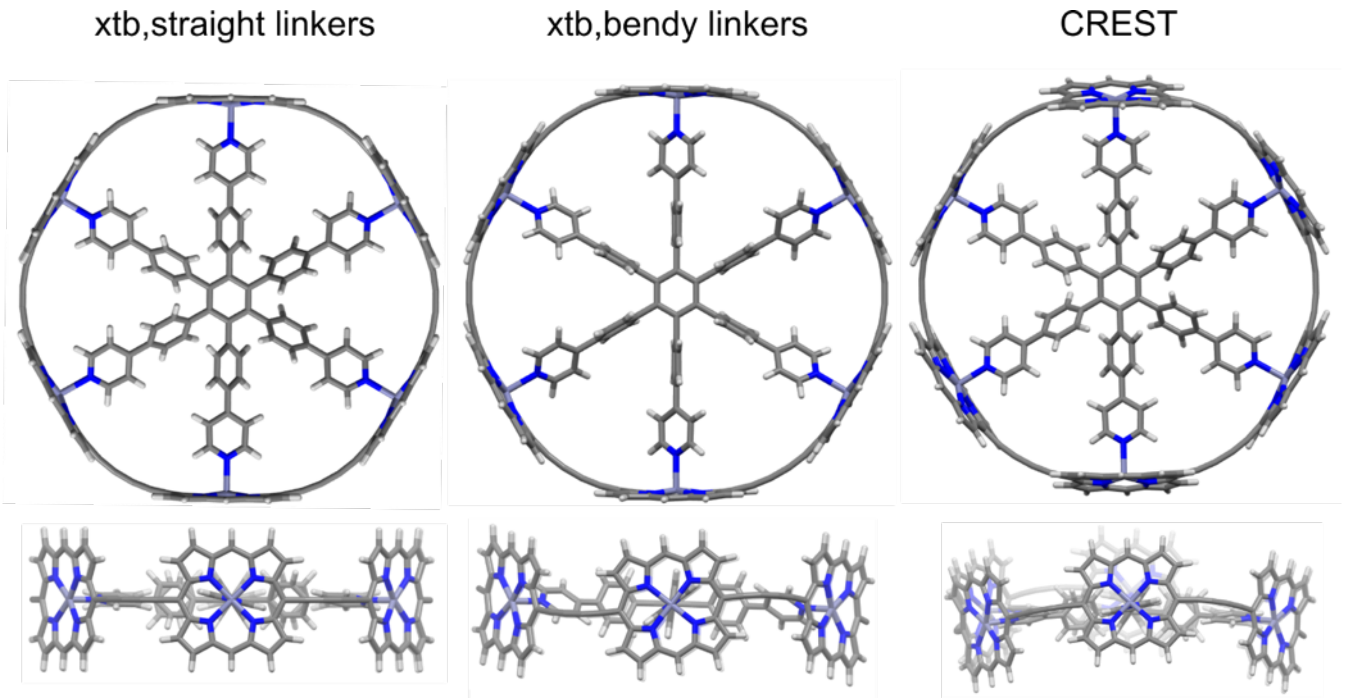

Figure S39. Overview of the xtb/CREST optimized structures.

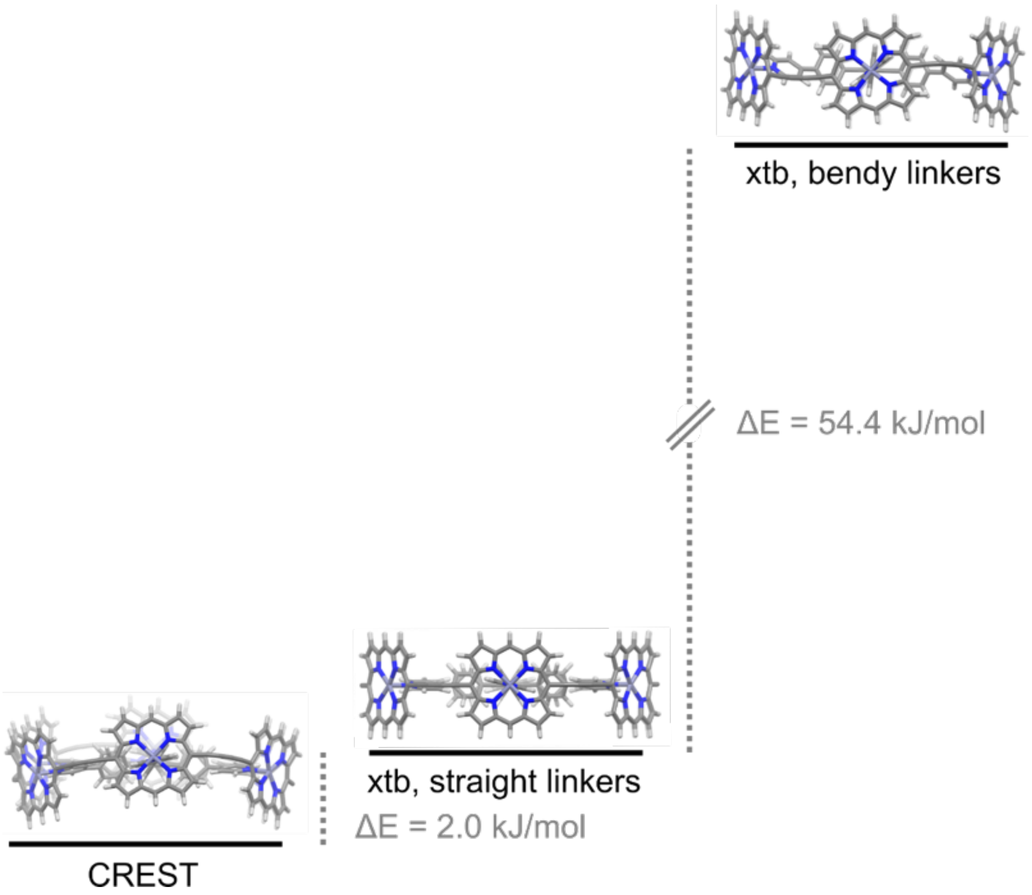

Figure S40. Energy diagram based on the performed xtb and CREST calculations.

The geometry obtained from CREST calculations is similar (with respect to the bendiness of the linkers) to a reported structure calculated on a CAM-B3LYP 6-31G(d) level of theory containing phenyl substituents in the porphyrin *meso* positions.[9] However, when aryl groups were deleted from this structure and substituted with hydrogen atoms and geometry optimization was repeated on the same level of theory, the geometry optimized to one with straight butadiyne linkers (Fig. S41), demonstrating that even a small change in molecular structure of **c-P6·T6** leads to a significantly different energy minimum. Frequency calculations were not performed for the DFT optimized structures due to their large size and therefore, significant computational resources and amount of time needed to complete the calculations.

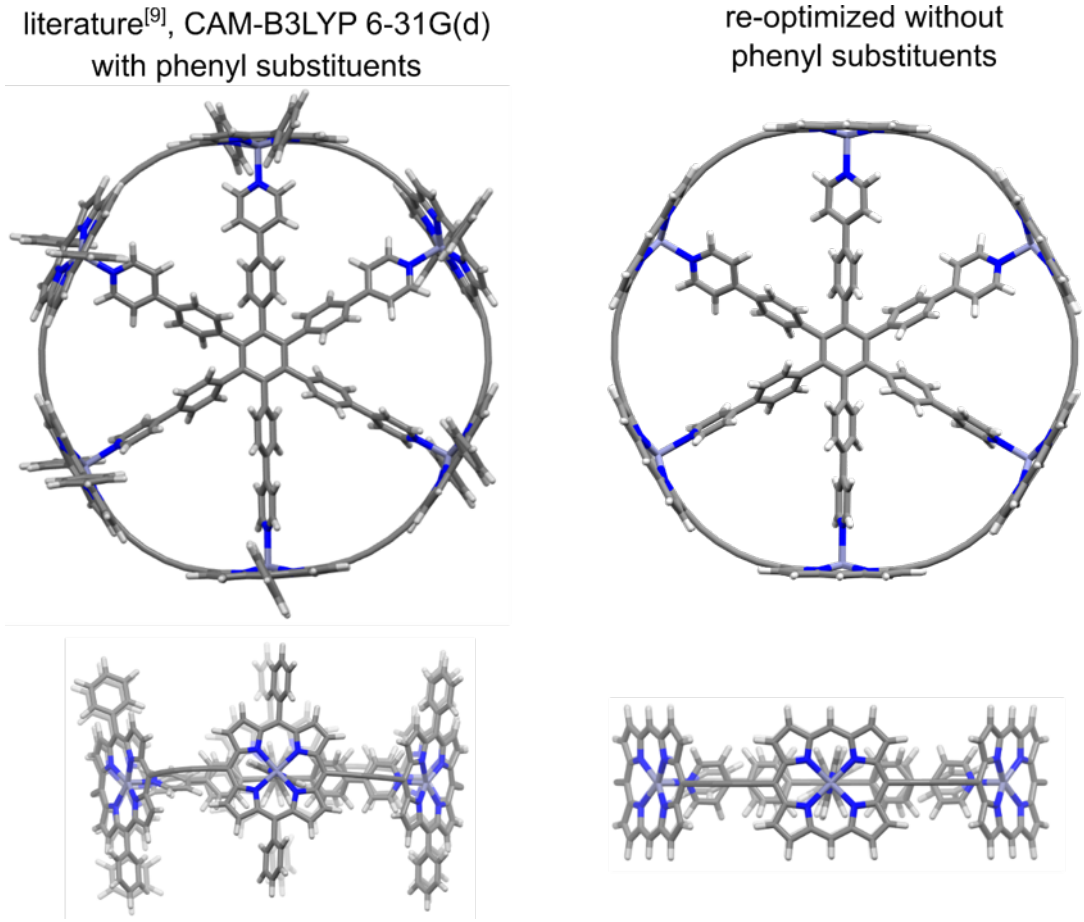

Figure S41. Two orthogolnal views on the CAM-B3LYP 6-31G(d) structure optimized with attached porphyrin *meso*-phenyl substituents[9] (left) and re-optimized structure (the same level of theory) after substituting the phenyl rings with hydrogen atoms (right).

The bendy and straight structures obtained from xtb calculations were subjected to geometry optimizations using DFT calculations on a B3LYP 6-31G(d,p) level of theory,[12] using Gaussian16 software.[13] The bendy structure underwent substantial structural changes during the optimization and converged to a geometry with straight butadiyne linkers, although the shape of the template fragment template is different and as a result, the energy difference on this level of theory is 13.4 kJ/mol in favor of the geometry obtained by starting from the straight structure with more propeller-shaped spokes of the template molecule. It reflects how difficult is it to perform calculations for such systems due to multiple possible relative geometries of the fragments and highlighting how important it is to get experimental X-ray diffraction data to discuss such complicated molecules in detail.

Next, we decided to compare energies using single-point calculations on different levels of theory, including those employed in the literature (e.g. LC-ωHPBE with $\omega = 0.1$, CAM-B3LYP 6-31G(d), BLYP35 6-31G(d)) and higher basis set (B3LYP def2-tzvp). We performed these calculations on three geometries:

- bendy geometry taken from xtb optimization,

- straight geometry taken from xtb calculations after re-optimization with B3LYP 6-31G(d,p),

- geometry from CREST conformational search.

The table S3 summarizes the obtained results.

STUCTURE	LEVEL	B3LYP 6-31G(d,p)	BLYP35 6-31G(d)	CAM-B3LYP 6-31G(d)	LC-wHPBE 6-31G(d) w=0.1	B3LYP def2-tzvp
**straight xtb**	x	-54121131.89	-54105610.27	-54107228.84	-54089231.12	-54134422.63
**bendy xtb**	x	-54120917.96	-54105492.15	-54107140.42	-54088929.28	-54134313.37
**CREST**	x	-54120911.33	-54105483.44	-54107137.91	-54088942.64	-54134292.57

Table S3. Results of the single-point calculations. Energy values are given in kJ/mol. Lowest-energy structures for each level theory are highlighted in gray.

The performed single-point calculations of theory show that on all tested levels of theory the straight geometry is the lowest-energy minimum and that the energy difference between this minimum and the next one (bendy geometry from xtb) is between 297 kJ/mol (LC-ωHPBE 6-31G(d) with $\omega = 0.1$) to 88 kJ/mol (CAM-B3LYP 6-31G(d)).

Based on these results, it is apparent that the geometry with straight linkers, previously not observed crystallographically and observed in our new crystal structures **P6-II** and **P6-III**, is the lowest energy minimum of all considered variants in the gas phase. It is always difficult to judge the validity of computational results and therefore, the data from X-ray studies are very valuable, because otherwise bent geometries would probably be discarded from consideration as they are very highly energetic, but accessible in the solid state.

6.2. ***c*-P6-free**

Geometry of the free-base six-porphyrin nanoring was optimized using xtb, starting with the geometry from the crystal structure **P6-C_{60}**. Aryl groups were omitted to simplify the calculations. The structure was optimized to meet standard convergence criteria, and the existence of a local minimum was verified by frequency calculation (--hess command). Negative frequencies were not observed.

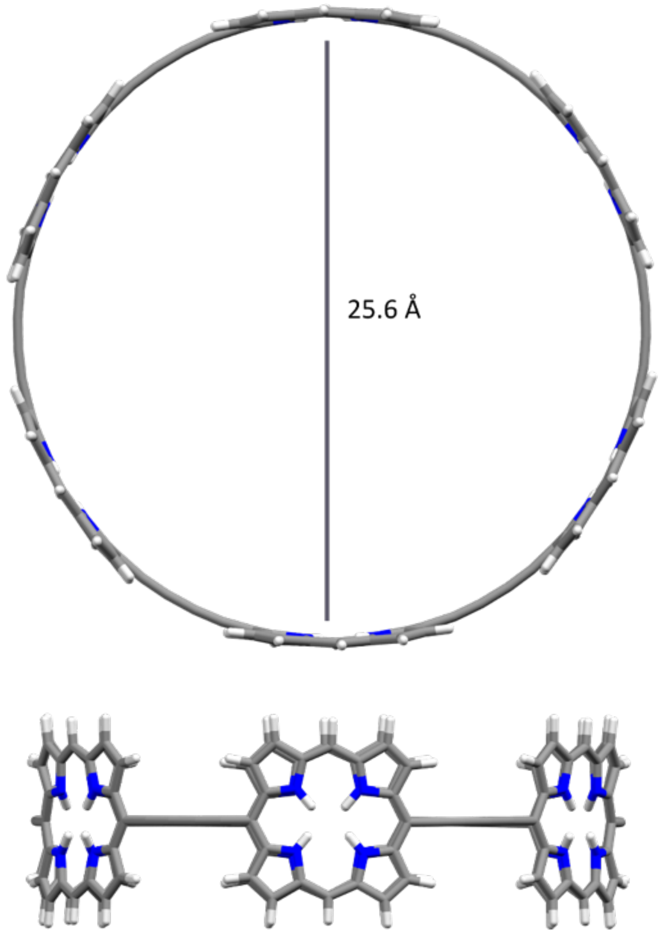

Figure S42. Two orthogonal views on the xtb-optimized structure of the metal-free nanoring with centroid-to-centroid distance between two opposite porphyrins.

All optimized geometries, conformers from CREST search and output files during frequency calculations for xtb models are available in Zenodo public repository: https://zenodo.org/doi/10.5281/zenodo.13134366.

7. References

[1] J. K. Sprafke, D. V. Kondratuk, M. Wykes, A. L. Thompson, M. Hoffmann, R. Drevinskas, W.-H. Chen, C. K. Yong, J.. Kärnbratt, J. E. Bullock, M. Malfois, M. R. Wasielewski, B. Albinsson, L. M. Herz, D. Zigmantas, D. Beljonne and H. L. Anderson, Belt-shaped π-systems: relating geometry to electronic structure in a six-porphyrin nanoring. *J. Am. Chem. Soc.,* 2011, **133**, 17262–17273.

[2] G. M. Sheldrick, SHELXT – integrated space-group and crystal-structure determination. *Acta Crystallogr.,* 2015, **A71**, 3–8.

[3] G. M. Sheldrick, Crystal structure refinement with SHELXL. *Acta Crystallogr.,* 2015, **C71**, 3–8.

[4] O. V. Dolomanov, L. J. Bourhis, R. J. Gildea, J. A. K. Howard and H. Puschmann, OLEX2: a complete structure solution, refinement and analysis program. *J. Appl. Crystallogr.,* 2009, **42**, 339–341.

[5] C. H. Devillers, A. K. D. Dimé, H. Cattey and D. Lucas, Crystallographic, spectroscopic and electrochemical characterization of pyridine adducts of magnesium(II) and zinc(II) porphine complexes. Comptes Rendus. Chimie 2013, **15**, 540-549.

[6] D. Kratzert and I. Crossing, Recent improvements in DSR. *J. Appl. Cryst.* 2018, **51**, 928-934.

[7] C. F. Macrae, I. Sovago, S. J. Cottrell, P. T. A. Galek, P. McCabe, M. Platings, G. P. Shields, J. S. Stevens, M. Towler and P. A. Wood, Mercury 4.0: from visualization to analysis, design and prediction. *J. Appl. Crystallogr.* 2020, **53**, 226–235.

[8] J.-R. Deng, D. Bradley, M. Jirásek, H. L. Anderson and M. D. Peeks, Correspondence on 'How aromatic are six-porphyrin nanorings? The case of a six-porphyrin nanoring'. *Angew. Chem. Int. Ed.*, 2022, **61**, e202201231.

[9] D. Bradley, M. Jirasek, H. L. Anderson and M. D. Peeks, Disentangling global and local ring currents, *Chem. Sci.* 2023, **14**, 1762–1768.

[10] P. Pracht, F. Bohle and S. Grimme, *Phys. Chem. Chem. Phys.* 2020, **22**, 7169–7192.

[11] C. Bannwarth, S. Ehlert and S. Grimme, GFN2-xTB-an accurate and broadly parametrized self-consistent tight-binding quantum chemical method with multipole electrostatics and density-dependent dispersion contributions, *J. Chem. Theory Comput.* 2019, **15**, 1652–1671.

[12] a) C. T. Lee, W. T. Yang and R. G. Parr, Development of the Colle-Salvetti Correlation-energy fFormula into a functional of the electron density. *Phys. Rev. B*, 1988, **37**, 785-789; b) A. D. Becke, Density-functional exchange-energy approximation with correct asymptotic behavior. *Phys. Rev. A* 1988, ***38***, 3098–3100.

[13] Gaussian 16, Revision C.01, M. J. Frisch, G. W. Trucks, H. B. Schlegel, G. E. Scuseria, M. A. Robb, J. R. Cheeseman, G. Scalmani, V. Barone, G. A. Petersson, H. Nakatsuji, X. Li, M. Caricato, A. V. Marenich, J. Bloino, B. G. Janesko, R. Gomperts, B. Mennucci, H. P. Hratchian, J. V. Ortiz, A. F. Izmaylov, J. L. Sonnenberg, D. Williams-Young, F. Ding, F. Lipparini, F. Egidi, J. Goings, B. Peng, A. Petrone, T. Henderson, D. Ranasinghe, V. G. Zakrzewski, J. Gao, N. Rega, G. Zheng, W. Liang, M. Hada, M. Ehara, K. Toyota, R. Fukuda, J. Hasegawa, M. Ishida, T. Nakajima, Y. Honda, O. Kitao, H. Nakai, T. Vreven, K. Throssell, J. A. Montgomery, Jr., J. E. Peralta, F. Ogliaro, M. J. Bearpark, J. J. Heyd, E. N. Brothers, K. N. Kudin, V. N. Staroverov, T. A. Keith, R. Kobayashi, J. Normand, K. Raghavachari, A. P. Rendell, J. C. Burant, S. S. Iyengar, J. Tomasi, M. Cossi, J. M. Millam, M. Klene, C. Adamo, R. Cammi, J. W. Ochterski, R. L. Martin, K. Morokuma, O. Farkas, J. B. Foresman, and D. J. Fox, Gaussian, Inc., Wallingford CT, 2016.
